# Synthesis of Nonplanar Graphene Nanoribbon with Fjord Edges

**DOI:** 10.1021/jacs.1c01882

**Published:** 2021-04-07

**Authors:** Xuelin Yao, Wenhao Zheng, Silvio Osella, Zijie Qiu, Shuai Fu, Dieter Schollmeyer, Beate Müller, David Beljonne, Mischa Bonn, Hai I. Wang, Klaus Müllen, Akimitsu Narita

**Affiliations:** † Max Planck Institute for Polymer Research, Ackermannweg 10, 55128 Mainz, Germany; ‡ Chemical and Biological Systems Simulation Lab, Center of New Technologies, University of Warsaw, 02-097 Warsaw, Poland; § Department of Chemistry, Johannes Gutenberg University Mainz, Duesbergweg 10-14, 55128 Mainz, Germany; ∥ Laboratory for Chemistry of Novel Materials, Université de Mons, B-7000 Mons, Belgium; ⊥ Institute for Physical Chemistry, Johannes Gutenberg University Mainz, Duesbergweg 10-14, 55128 Mainz, Germany; # Organic and Carbon Nanomaterials Unit, Okinawa Institute of Science and Technology Graduate University, Okinawa 904-0495, Japan

## Abstract

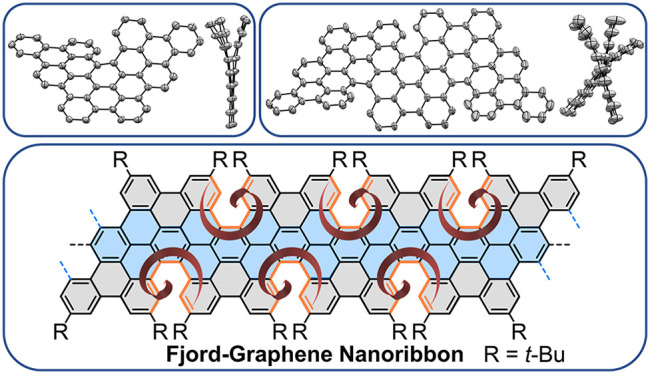

As a new family of semiconductors, graphene nanoribbons (GNRs), nanometer-wide strips of graphene, have appeared as promising candidates for next-generation nanoelectronics. Out-of-plane deformation of π-frames in GNRs brings further opportunities for optical and electronic property tuning. Here we demonstrate a novel fjord-edged GNR (**FGNR**) with a nonplanar geometry obtained by regioselective cyclodehydrogenation. Triphenanthro-fused teropyrene **1** and pentaphenanthro-fused quateropyrene **2** were synthesized as model compounds, and single-crystal X-ray analysis revealed their helically twisted conformations arising from the [5]helicene substructures. The structures and photophysical properties of **FGNR** were investigated by mass spectrometry and UV–vis, FT-IR, terahertz, and Raman spectroscopic analyses combined with theoretical calculations.

Graphene nanoribbons (GNRs), quasi-one-dimensional (1D) cutouts of graphene, have been highlighted as promising candidates for next-generation electronics because their intriguing electronic properties can be precisely tuned by their chemical structures.^1−5^ The development of cutting-edge synthetic methods, especially bottom-up synthesis,^6−8^ utilizing on-surface and solution-mediated reactions, has delivered structurally defined GNRs with various edge structures, including armchair,^9−11^ zigzag,^12^ cove,^13^ and gulf.^14^ Furthermore, the peripheral features significantly alter the electronic properties of GNRs. The emergence of exotic topological electronic states in a GNR with hybrid edge structures combining the armchair and zigzag may serve as a typical example.^15^

On the other hand, in addition to GNRs with planar conformations, GNRs with nonplanar geometries featuring out-of-plane-distorted π-frames are expected to provide further opportunities in, for example, nonlinear optics,^16,17^ nanomechanics,^18^ and asymmetric catalysis.^19^ However, the synthesis of nonplanar GNRs has been relatively underexplored.^20^ In 2015, we synthesized a cove-edged GNR with a chrysene-based structure on-surface. This cove-edged GNR featured a unique nonplanar up–down geometry resulting from [4]helicene motifs, as revealed by crystallographic characterization of their oligomer synthesized in solution.^13^

Significant efforts have been made to achieve nonplanar GNRs with larger distortions. In 2020, short segments of fjord-edged GNRs with a remarkably twisted conformation were independently reported by the groups of Campaña^21^ and Wang^22^ through symmetrical incorporation of [5]helicene motifs into peripheral sites, although their extensions to long GNRs were not described. On the other hand, Liu, Mai, and colleagues demonstrated a solution synthesis of the first example of long curved GNRs featuring a combination of cove, zigzag, and armchair edges.^23^ Very recently, Rubin and co-workers reported the synthesis of a nitrogen-doped fjord-edged GNR via solid-state topochemical polymerization, although its nonplanarity was not discussed and the resulting GNRs seemed to have only limited solubility.^24^ Thus, the development of highly twisted fjord-edged GNRs in solution has remained elusive.

Herein we describe an efficient solution synthesis of a novel fjord-edged GNR (**FGNR**) via AB-type Suzuki polymerization^25^ followed by a regioselective Scholl reaction. As subunits of **FGNR**, two significant model compounds were synthesized: triphenanthro-fused teropyrene **1** and pentaphenanthro-fused quateropyrene **2** (Scheme 1). Bulky *tert*-Butyl substituents were appropriately installed on the respective precursor molecules **5** and **9** to prohibit their complete cyclodehydrogenation during the Scholl reaction,^21,22,26^ leading to the unsymmetrical incorporation of [5]helicene substructures along the peripheral sites of **1** and **2**. Crystallographic analyses of **1** and **2** clearly elucidated their helically twisted geometries, which suggests similar nonplanar conformation for the corresponding **FGNR**. The successful formation of **FGNR** via the optimized Scholl reaction was corroborated by Fourier transform infrared (FT-IR) and Raman spectroscopy and matrix-assisted laser desorption/ionization time-of-flight mass spectrometry (MALDI-TOF MS). Besides, time-resolved terahertz spectroscopy revealed an intrinsic charge-carrier mobility of >100 cm^2^·V^–1^·s^–1^ in the **FGNR**, which suggests its potential for applications in electronic devices.

**Scheme 1:**
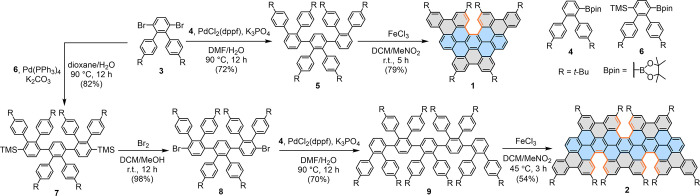
Synthetic Routes toward Model Compounds **1** and **2**

The synthesis of **1** and **2** as model compounds is depicted in Scheme 1. We designed oligophenylene precursors **5** and **9** bearing *tert*-butyl groups that can enable the selective cyclodehydrogenation reaction (as demonstrated in 2011 by the group of Durola^26^). First, oligophenylene **5** was synthesized via Suzuki coupling of 3′,6′-dibromo-4,4″-di-*tert*-butyl-1,1′:2′,1″-terphenyl (**3**) with terphenyl boronic ester **4**. Then the Scholl reaction of **5** with iron(III) chloride (FeCl_3_) at room temperature selectively gave **1** in 79% yield. Toward the synthesis of **2**, oligophenylene **8** with two bromo substituents was initially prepared via twofold Suzuki coupling of **3** with *o*-terphenyl boronic ester **6** followed by conversion of the trimethylsilyl substituents to bromo groups. Subsequently, Suzuki coupling of **8** with **4** afforded oligophenylene precursor **9** in 70% yield. The initially attempted Scholl reaction of **9** with FeCl_3_ at room temperature failed to give the targeted **2** but yielded a mixture of products (Figure S1). Unsatisfying results were also found when 2,3-dichloro-5,6-dicyano-1,4-benzoquinone (DDQ)/trifluoromethanesulfonic acid (CF_3_SO_3_H) was used as the Lewis oxidant/acid combination. To our delight, **2** was obtained in 54% yield by refluxing a solution of **9** in unstabilized dichloromethane (DCM) in the presence of FeCl_3_ (see synthetic details in the Supporting Information (SI)).

The formation of **1** and **2** was first validated by high-resolution MALDI-TOF MS (Figure S2). Thanks to the good solubility, the structure of **1** was further characterized by ^1^H, ^13^C, and 2D NMR measurements, in which the well-resolved proton signals could be fully assigned (see the NMR spectra in SI). In contrast, a complex spectrum with considerable signal overlaps was recorded for **2**, which could be rationalized by the three unsymmetrically incorporated [5]helicene subunits, resulting in multiple diastereomers with relatively large isomerization barriers (>36 kcal/mol; Figure S3).

Single crystals of **1** and **2** were obtained by slow evaporation of their solutions in DCM (or CD_2_Cl_2_), allowing X-ray diffraction analysis to be performed (Figure 1). **1** and **2** adopt helically twisted conformations resulting from strong steric repulsion of the bulky *tert*-butyl groups. Remarkably, because of the unsymmetrical arrangement of [5]helicene motifs, **2** exhibits discernible end-to-end twists of 83° and 50°. For both **1** and **2**, two enantiomers ([*M*]- and [*P*]-**1** and [*M*,*M*,*M*]- and [*P*,*P*,*P*]-**2**, respectively) are present in a 1:1 ratio in the unit cell with edge-to-edge π–π interactions (Figure 1c,d). Chiral high-performance liquid chromatography (HPLC) separation of **1** afforded two isolated fractions (Figure S4a), which demonstrated a mirror-symmetrical pattern in circular dichroism (CD) spectroscopy (Figure S4b). However, overlapping peaks in the chiral HPLC chart of **2** arising from multiple enantiomers made the separation unsuccessful (Figure S5).

**Figure 1:**
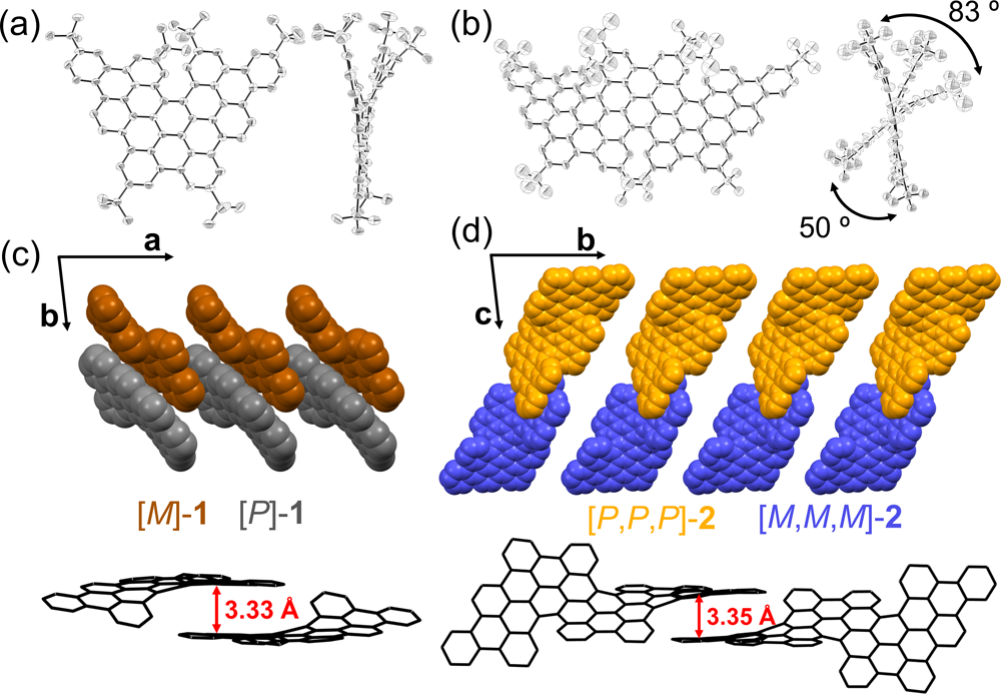
Crystal structures of **1** and **2**. (a, b) ORTEP drawing of **1** and **2**, with thermal ellipsoids shown at 50% probability. (c, d) Crystal-packing structures of **1** and **2**. Hydrogen atoms, *tert*-butyl groups in (c) and (d), and solvent molecules have been omitted for clarity.

For the synthesis of **FGNR**, *o*-terphenyl **10** functionalized with bromo and boronic ester substituents was synthesized through selective monolithiation/borylation of **3** (Scheme 2). Subsequently, AB-type Suzuki polymerization of **10** gave *tert*-butylphenyl-substituted poly(*p*-phenylene) precursor **P1**. After Soxhlet extraction with acetone, analysis of **P1** by size-exclusion chromatography (SEC) with polystyrene as a standard indicated a number-average molecular weight (*M*_n_) of around 1.5 × 10^4^ g mol^–1^ and a polydispersity index (PDI) of 2.4 (Figure S6). On the other hand, MALDI–TOF MS analysis of **P1** revealed a periodic pattern of signals up to *m*/*z* ≈ 15 000 with gaps of *m*/*z* ≈ 340, in agreement with the molecular mass of the repeating unit (Figure S7). Finally, the Scholl reaction of **P1** with FeCl_3_ (5.6 equiv per H to be removed) in unstabilized DCM at reflux for 48 h afforded **FGNR** as a dark-red solid. The estimated length of **FGNR** is ∼17 nm based on the SEC results for **P1**.

**Scheme 2:**
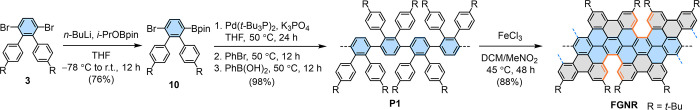
Schematic Illustration of the Synthesis of **FGNR**

FT-IR spectroscopic analysis of the model compounds and **FGNR** was performed with the support of density functional theory (DFT) calculations (Figure 2a). Distinct C–H out-of-plane (*opla*) modes arising from the different edge structures are clearly elucidated, showing general agreement between the experimental and theoretical spectra. **1**, **2**, and **FGNR** are characterized by the so-called SOLO mode (wagging of an isolated aromatic C–H bond neighbored by two C–C bonds)^27^ at 870 cm^–1^ (orange-colored in Figure 2a), which is absent in the spectrum of **P1** (see the SI). Comparing the spectra of **1**, **2**, and **FGNR**, the intensity of the DUO mode (wagging of two adjacent aromatic C–H bonds)^27^ at 820 cm^–1^ (purple-colored in Figure 2a) gradually decreases, in agreement with the fact that the DUO mode arises only from the ends of **FGNR**. Remarkably, the observation of the SOLO mode at around 660 cm^–1^ (green-colored in Figure 2a), which can be assigned to the [5]helicene substructures, corroborates the fjord edge structure in **1**, **2**, and **FGNR**.

**Figure 2:**
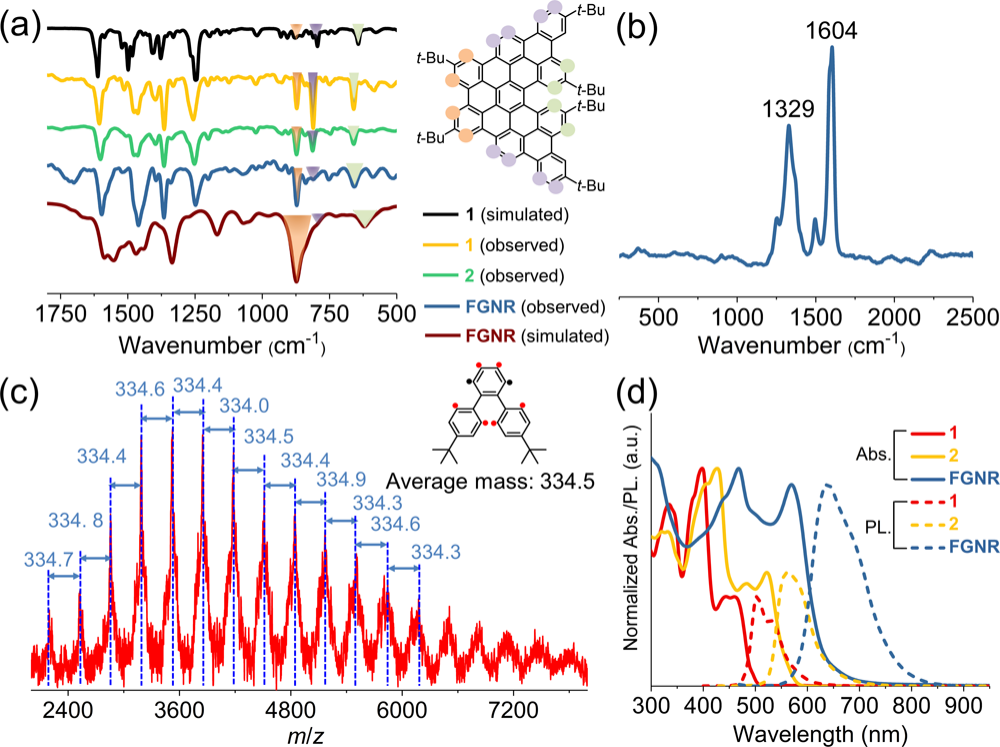
(a) FT-IR spectra of **1**, **2**, and **FGNR** measured on powder samples and DFT-calculated spectra of **1** and **FGNR** at the HSE06/6-31G(d) level. (b) Raman spectrum of **FGNR** recorded with a 488 nm excitation laser (baseline subtraction was carried out because of the strong fluorescence background of **FGNR**). (c) MALDI-TOF MS analysis of **FGNR** (matrix: tetracyanoquinodimethane, linear mode). (d) UV–vis and photoluminescence spectra of **1** and **2** (10^–5^ M) and **FGNR** (0.1 mg/mL) in THF.

The successful formation of **FGNR** was further supported by Raman spectroscopy (Figure 2b). Two main peaks are found at 1604 and 1329 cm^–1^, which can be assigned as the G and D peaks, respectively, similar to previous reports on GNRs.^28^ Moreover, MALDI-TOF MS analysis of **FGNR** demonstrated a sequence of peaks with an interval of *m*/*z* ≈ 334 (Figure 2c), consistent with the loss of six protons from each repeating unit of **P1** during the Scholl reaction. The efficiency of cyclodehydrogenation via the Scholl reaction was estimated to be 97% according to the mass difference between the MALDI-TOF MS results for **FGNR** and **P1** (Table S1).^9^

The UV–vis absorption and photoluminescence spectra of **FGNR** and model compounds **1** and **2** were recorded in tetrahydrofuran (THF) (Figure 2d). The longest absorption band appears at 461 and 522 nm for **1** and **2**, respectively, and is assignable to the highest occupied molecular orbital (HOMO) to lowest unoccupied molecular orbital (LUMO) electronic transition according to time-dependent density functional theory (TD-DFT) calculations (see the SI). **FGNR** with the extended π-conjugation displayed a red-shifted absorption peak at 569 nm. The optical energy gaps of **1** and **2** deduced from the absorption onset are 2.52 and 2.25 eV, respectively. Likewise, an optical energy gap of 1.99 eV is estimated for **FGNR**, which is in line with the calculated result (1.93 eV) and comparable to the value reported for a fjord-edge nitrogen-doped GNR (2.04 eV).^24^ Photoluminescence spectra of **1**, **2**, and **FGNR** display a red shift of the emission maxima following the extension of the π-conjugation. In particular, broad emission covering 550–850 nm is found for **FGNR**, which could potentially be interesting for optoelectronic device applications.

To investigate the charge-carrier transport properties of **FGNR**, we measured its photoconductivity using ultrafast optical pump–terahertz probe (OPTP) spectroscopy. Figure 3a displays the time-resolved complex photoconductivity dynamics of **FGNR** dispersed in 1,2,4-trichlorobenzene following optical excitation by 400 nm laser pulses. The subpicosecond rise of the real conductivity represents the photogeneration of initially free carriers, and the subsequent rapid decay can be attributed to the formation of excitons, i.e., bound electron–hole pairs (featured by a large imaginary conductivity plus a small real conductivity on a longer time scale).^29,30^ To quantify free charge carriers’ transport properties at early delay times, we recorded the conductivity spectrum at ∼1.2 ps after photoexcitation, as shown in Figure 3b. The frequency-resolved complex conductivity can be well-described by the Drude–Smith (DS) model (for details, see the SI).^31,32^ The DS model assumes bandlike transport, where charge scattering occurs not randomly but preferentially through backscattering due to, e.g., conjugation or torsional defects in the materials. A parameter *c* characterizes the backscattering probability, which ranges between 0 (isotropic scattering) and −1 (100% backscattering). From the fit, we obtained a charge scattering time τ = 28 ± 1 fs and *c* = −0.99 ± 0.01. The inferred *c* value of −0.99 ± 0.01 indicates nearly full backscattering of charge carriers in **FGNR**. Since the length of the ribbons (17 nm) is comparable to the mean free propagation path of charge carriers in GNRs (10–20 nm),^33^ the backscattering effect in **FGNR** likely originates from scattering at the ends of the ribbons. Furthermore, using an effective charge carrier mass m* = 0.48*m*_0_ (considering contributions from both electrons and holes; see the SI for details) and τ from the DS fit, we estimate the intrinsic charge mobility μ (=*e*τ/m*) to be 104 ± 3 cm^2^·V^–1^·s^–1^, which is sufficiently high for electrical devices.

**Figure 3:**
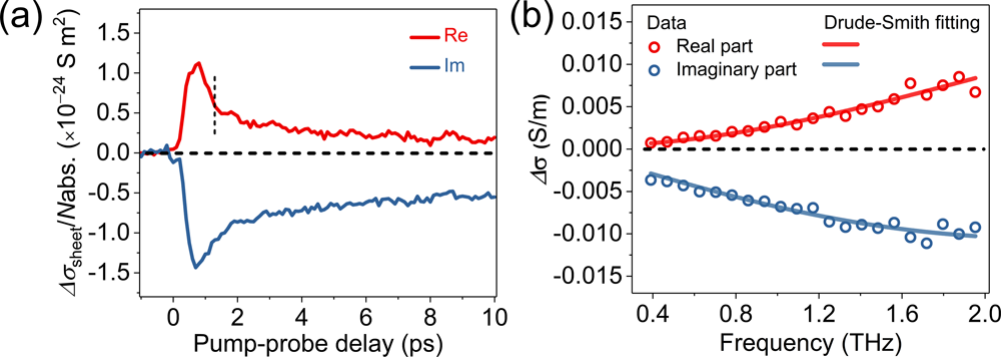
(a) Time-resolved complex terahertz photoconductivity of **FGNR** normalized to the absorbed photon density. (b) Frequency-resolved terahertz conductivity measured at ∼1.2 ps after photoexcitation (denoted by the dashed vertical line in (a)). The solid lines are fits to the Drude–Smith model.

In summary, we have demonstrated an efficient approach toward novel oligomeric and polymeric helically twisted GNRs featuring a fjord-type periphery via a regioselective Scholl reaction. Helically twisted conformations were unambiguously revealed for model compounds **1** and **2** by crystallographic characterizations, suggesting a similar conformation of **FGNR**. A photoconductivity investigation of **FGNR** via terahertz spectroscopy indicated an intrinsic charge-carrier mobility of approximately 100 cm^2^·V^–1^·s^–1^, rendering this **FGNR** a candidate for nanoelectronic devices. This study features a new synthetic strategy based on regioselective cyclodehydrogenation, which can potentially be applied to a variety of fjord-edged GNRs with nonplanar structures. Considering the chirality originating from the fjord periphery, such GNRs could further provide an entry to technological applications utilizing chiroptical responses.

## Supporting Information Available

Experimental details; Scheme S1, Figures S1–S49, Tables S1–S3; and calculations on **1**, **2**, and **FGNR** (PDF)

## Accession Codes

CCDC 2058017 and 2058018 contain the supplementary crystallographic data for this paper. These data can be obtained free of charge via www.ccdc.cam.ac.uk/data_request/cif, or by emailing data_request@ccdc.cam.ac.uk, or by contacting The Cambridge Crystallographic Data Centre, 12 Union Road, Cambridge CB2 1EZ, U.K.; fax: +44 1223 336033.
