# Supplementary material for: Synthesis of Nonplanar Graphene Nanoribbon with Fjord Edges
Source: J Am Chem Soc. 2021 Apr 7;143(15):5654–8. doi: 10.1021/jacs.1c01882 (PMC8154539; doi:10.1021/jacs.1c01882)
Supplement: Supplementary file 1 — ja1c01882_si_001.pdf [file ja1c01882_si_001.pdf]

Supporting Information

Synthesis of Non-Planar Graphene Nanoribbon with Fjord Edges

Xuelin Yao,[a] Wenhao Zheng,[a] Silvio Osella,[b] Zijie Qiu,[a] Shuai Fu,[a] Dieter Schollmeyer,[c] Beate Müller,[a] David Beljonne,[d] Mischa Bonn,[a] Hai I. Wang,[a] Klaus Müllen,[a,e*] and Akimitsu Narita[a,f*]

[a] Max Planck Institute for Polymer Research, Ackermannweg 10, 55128 Mainz, Germany

[b] Chemical and Biological Systems Simulation Lab, Center of New Technologies, University of Warsaw, 02-097 Warsaw, Poland

[c] Department of Chemistry, Johannes Gutenberg University Mainz, Duesbergweg 10-14, 55128 Mainz, Germany

[d] Laboratory for Chemistry of Novel Materials, Université de Mons, B-7000 Mons, Belgium

[e] Institute for Physical Chemistry, Johannes Gutenberg University Mainz, Duesbergweg 10-14, 55128 Mainz, Germany

[f] Organic and Carbon Nanomaterials Unit, Okinawa Institute of Science and Technology Graduate University, Okinawa 904-0495, Japan

Content

1. General methods and materials

2. Synthetic procedures

3. Mass spectra

4. Isomerization processes of **1** and **2**

5. Optical resolution by HPLC

6. SEC and MALDI-TOF analysis of **P1**

7. FT-IR spectrum

8. Electrochemical properties of **1** and **2**

9. X-ray crystallographic analysis of **1** and **2**

10. DFT calculations

11. Terahertz spectroscopic study of **FGNR**

12. NMR spectra

13. References

1. General methods and materials

All commercially available chemicals were used without further purification unless otherwise noted. Column chromatography was conducted with silica gel (grain size 0.04 – 0.063 mm) and thin layer chromatography (TLC) was performed on silica gel-coated aluminium sheets with F254 indicator. Nuclear magnetic resonance (NMR) spectra were recorded on Bruker Avance 300, 500 or 850 MHz spectrometer. Chemical shifts were reported in ppm. Coupling constants (*J* values) were presented in Hertz (Hz). ^{1}H NMR chemical shifts were referenced to CD_2Cl_2 (5.32 ppm) or $C_2D_2Cl_4$ (6.00 ppm). ^{13}C NMR chemical shifts were referenced to CD_2Cl_2 (53.84 ppm) or $C_2D_2Cl_4$ (73.78 ppm). Abbreviations: s =singlet, d =doublet, dd =double doublet, t =triplet, m = multiplet, td = triplet of doublets. High-resolution mass spectrometry (HRMS) was performed on a SYNAPT G2 Si high resolution time-of-flight (TOF) mass spectrometer (Waters Corp., Manchester, UK) by matrix-assisted laser desorption/ionization (MALDI). Analytical size-exclusion chromatography (SEC) was performed on SDV PSS GPC columns using tetrahydrofuran (THF) as eluent at a temperature of 303 K. Fourier-transform infrared spectroscopy (FT-IR) measurement was conducted with Bruker Tensor II FTIR spectrometer. Raman spectrum was measured with a Bruker RFS 100/S Raman spectrometer. Melting points were measured with a Büchi B-545 apparatus. Absorption spectra were recorded on a Perkin-Elmer Lambda 900 spectrophotometer. Photoluminescence spectra were recorded on a J&M TIDAS spectrofluorometer. Photoluminescence spectra of **1**, **2**, and **FGNR** were recorded via exciting at their absorption maxima. High performance liquid chromatography (HPLC) analysis was performed on an Agilent 1200 Series equipped with the following modules: quaternary pump (G1311A 1100), Manual Sample Injector (Rheodyne 7725i), column thermostat (G1316A 1200) and DAD detector (G1713B 1200). For reversed phase HPLC, column Gravity C8 (3 × 100 mm, 5µm) from Macherey-Nagel was used. The chiral column used was Daicel Chiralpak IE analytical column (4.6 × 250 mm) packed with amylose tris-(3,5-dichlorophenylcarbamate) immobilized on silica gel (5 µm). In both cases, the column temperature was set at 20 °C and the flow was constant during the operation.

Circular dichroism (CD) spectra were collected on JASCO J-1500 circular dichroism spectrometer at 298 K in anhydrous THF. Cyclic voltammetry (CV) was performed on a WaveDriver 20 Bipotentiostat/Galvanostat (Pine Instruments Company) and measurements were carried out in dichloromethane (anhydrous, HPLC grade) containing 0.1 M n-Bu_4NPF_6 as supporting electrolyte (scan rate: 100 mV s^{-1}). A glassy carbon electrode was used as a working electrode, a platinum wire as a counter electrode and a silver wire as a reference electrode.

Dibromo-1,4-bis(trimethylsilyl)benzene (**11**) and 1,2-dibromo-3-(trimethylsilyl)benzene (**13**) were prepared following reported procedures.[1]

2. Synthetic procedures

Scheme S1. Synthetic route to compounds **3**, **4** and **6**.

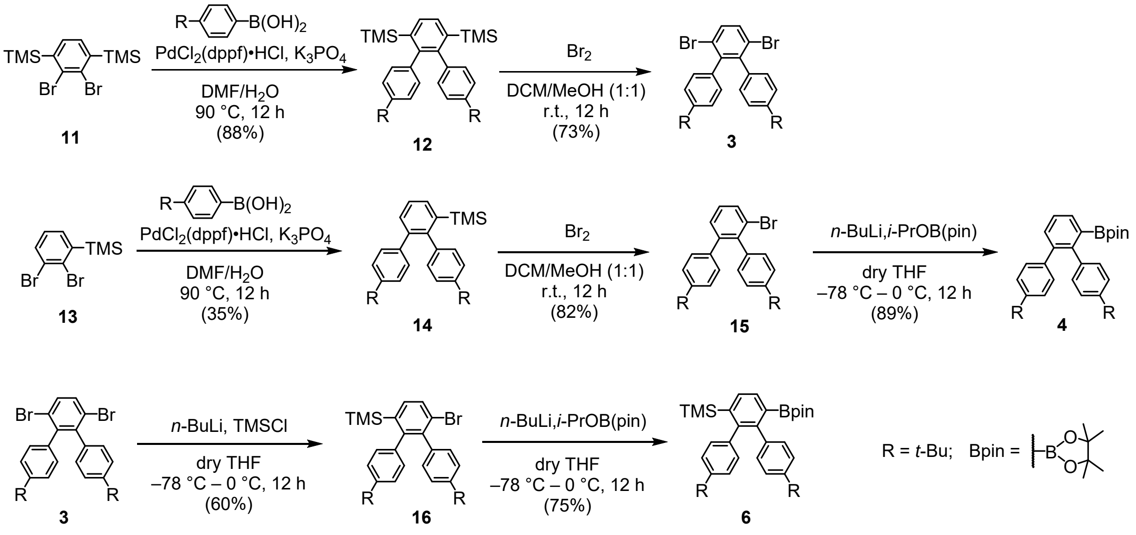

Synthesis of [4,4''-di-*tert*-butyl-(1,1':2',1''-terphenyl)-3',6'-diyl]bis(trimethylsilane) (12)

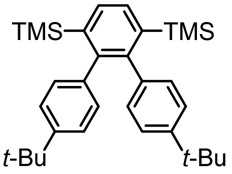

Dibromo-1,4-bis(trimethylsilyl)benzene (**11**) (2.00 g, 5.26 mmol), 4-*tert*-butylphenylboronic acid (4.68 g, 26.3 mmol), tripotassium phosphate (6.71 g, 31.6 mmol), and [1,1'-bis(diphenylphosphino) ferrocene]dichloropalladium(II) complex with dichloromethane (212 mg, 0.260 mmol) were dissolved in a mixture of dimethylformamide (50 mL) and water (10 mL). After degassing via bubbling through argon for 0.5 h, the resulting mixture was heated to 90 °C with stirring for 12 h. The reaction mixture was then cooled to room temperature, and 100 mL of water was added. The aqueous phase was extracted by diether ether (three times). The combined organic phases were washed with water and dried over magnesium sulfate. After the removal of the solvent under reduced pressure, the residue was purified by column chromatography over silica gel using *n*-hexane as eluent to give the title compound as a white solid (2.24 g, 88%). M.p.: 251.1 – 251.8 °C. ^{1}H NMR (300 MHz, CD_2Cl_2, 298 K, ppm) δ 7.59 (s, 2H), 7.07 (d, J = 8.2 Hz, 4H), 6.84 (d, J = 8.2 Hz, 4H), 1.21 (s, 18H), 0.00 (s, 18H); ^{13}C NMR (75 MHz, CD_2Cl_2, 298 K, ppm) δ 149.43, 147.96, 140.35, 139.87, 132.80, 131.02, 123.75, 34.56, 31.43, 0.51; HRMS (MALDI) *m/z*: Calcd for $C_{32}H_{46}Si_2$: 486.3138; Found: 486.3132 $[M]^+$.

Synthesis of 3',6'-dibromo-4,4''-di-*tert*-butyl-1,1':2',1''-terphenyl (3)

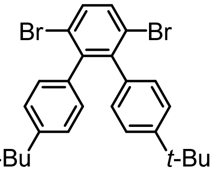

To a solution of compound **12** (1.71 g, 3.51mmol) in 40 mL of methanol and 40 mL of dichloromethane was added bromine (1.68 g, 10.5 mmol) dropwise. The mixture was stirred at room temperature for 12 h. Then, the reaction was quenched by saturated aqueous sodium sulfite solution. The aqueous phase was extracted by dichloromethane (three times). The combined organic phases were washed with water, and dried over magnesium sulfate. After the solvents were removed under reduced pressure, the residue was purified by column chromatography over silica gel using *n*-hexane as eluent to afford the title compound as a white solid (1.28 g, 73%). M.p.: 177.7 – 178.5 °C. ^{1}H NMR (300 MHz, CD_2Cl_2, 298 K, ppm) δ 7.54 (s, 2H), 7.14 (d, J = 8.0 Hz, 4H), 6.86 (d, J = 8.0 Hz, 4H), 1.22 (s, 18H); ^{13}C NMR (75 MHz, CD_2Cl_2, 298 K, ppm) δ 150.33, 144.71, 137.60, 132.93, 129.96, 124.50, 123.49, 34.69, 31.34; HRMS (MALDI) *m/z*: Calcd for $C_{26}H_{28}Br_2$: 498.0558; Found: 498.0574 $[M]^+$.

Synthesis of [4,4''-di-*tert*-butyl-(1,1':2',1''-terphenyl)-3'-yl]trimethylsilane (14)

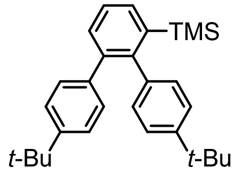

1,2-Dibromo-3-(trimethylsilyl)benzene (**13**) (10.0 g, 32.5 mmol), 4-*tert*-butylphenylboronic acid (23.1 g, 130 mmol), tripotassium phosphate (41.4 g, 195 mmol) and [1,1'-bis(diphenylphosphino)ferrocene]dichloropalladium(II) complex with dichloromethane (796 mg, 0.975 mmol) were dissolved in a mixture of dimethylformamide (150 mL) and water (30 mL). The mixture was degassed via bubbling through argon for 1 h. Then, the mixture was heated to 90 °C with stirring for 12 h. The resulting mixture was cooled to room temperature, followed by addition of 200 mL of water. The mixture was then extracted with diethyl ether (three times) and washed with water. The combined organic phases were dried over magnesium sulfate. After the solvents were removed under reduced pressure, the residue was purified by column chromatography over silica gel using *n*-hexane as eluent to afford the title compound as a white solid (4.71 g, 35%). M.p.: 127.5 – 128.4 °C. ^{1}H NMR (300 MHz, CD_2Cl_2, 298 K, ppm) δ 7.61 (dd, J = 6.8, 2.0 Hz, 1H), 7.43 – 7.29 (m, 2H), 7.25 – 7.08 (m, 4H), 7.05 – 6.91 (m, 4H), 1.27 (s, 9H), 1.23 (s, 9H), 0.00 (s, 9H); ^{13}C NMR (75 MHz, CD_2Cl_2, 298 K, ppm) δ 150.10, 149.09, 147.46, 141.54, 140.24, 139.70, 139.60, 133.81, 130.98, 129.84, 126.72, 124.50, 124.22, 118.53, 34.63, 34.51, 31.42, 31.34, 0.66; HRMS (MALDI) *m/z*: Calcd for $C_{29}H_{38}Si$: 414.2743; Found: 414.2717 $[M]^+$.

Synthesis of 3'-bromo-4,4''-di-*tert*-butyl-1,1':2',1''-terphenyl (15)

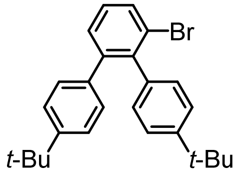

To a solution of compound **14** (2.34 g, 5.64 mmol) in 12 mL of methanol and 12 mL of dichloromethane, bromine (1.35 g, 8.46 mmol) was added dropwise. The mixture was stirred at room temperature for 12 h. The reaction was then quenched by saturated aqueous sodium sulfite solution. The aqueous phase was extracted by dichloromethane (three times). The combined organic phases were washed with water, dried over magnesium sulfate. After the removal of the solvent under reduced pressure, the residue was purified by column chromatography over silica gel using *n*-hexane as eluent to give the title compound as a white solid (1.95 g, 82%). M.p.: 85.5 – 86.1 °C. ^{1}H NMR (300 MHz, CD_2Cl_2, 298 K, ppm) δ 7.67

(d, J = 7.9 Hz, 1H), 7.37 (d, J = 7.6 Hz, 1H), 7.26 (dd, J = 8.0, 5.6 Hz, 3H), 7.14 (d, J = 8.0 Hz, 2H), 6.97 (dd, J = 17.5, 7.9 Hz, 4H), 1.29 (s, 9H) 1.24 (s, 9H); ^{13}C NMR (75 MHz, CD_2Cl_2, 298 K, ppm) δ 150.44, 149.91, 144.27, 141.62, 138.60, 137.69, 131.90, 130.62, 129.68, 129.64, 128.92, 124.91, 124.76, 124.73, 34.78, 34.63, 31.44, 31.37; HRMS (MALDI) *m/z*: Calcd for $C_{26}H_{29}Br$: 420.1453; Found: 420.1468 $[M]^+$.

Synthesis of 2-[4,4''-di-*tert*-butyl-(1,1':2',1''-terphenyl)-3'-yl]-4,4,5,5-tetramethyl-1,3,2-dioxaborolane (4)

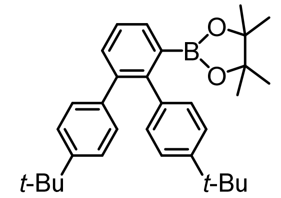

Compound **15** (1.61 g, 3.81 mmol) in 55 mL of anhydrous tetrahydrofuran at −78 °C was added *n*-BuLi (2.6 mL, 1.6 M in hexanes) dropwise. The yellow solution was stirred at −78 °C for 1 h. 2-Isopropoxy-4,4,5,5-tetramethyl-1,3,2-dioxaborolane (851 mg, 4.57 mmol) in 5 mL of anhydrous tetrahydrofuran was added dropwise into the solution. The resulting mixture was gradually warmed to room temperature and kept stirring for 3 h. The reaction mixture was then quenched by adding 30 mL of water. The aqueous phase was extracted by dichloromethane (three times). The combined organic phases were washed with water and dried over magnesium sulfate. After the removal of the solvent under reduced pressure, the residue was purified by column chromatography over silica gel using *n*-hexane/dichloromethane (3:1) as eluent to afford the title compound as a white solid (1.60 g, 89%). M.p.: 143.5 – 144.1 °C. ^{1}H NMR (300 MHz, CD_2Cl_2, 298 K, ppm) δ 7.58 (d, J = 6 Hz, 1H), 7.41 – 7.34 (m, 2H), 7.20 (dd, J = 8.4, 3.1 Hz, 4H), 7.03 – 6.97 (m, 4H), 1.29 (s, 9H), 1.27 (s, 9H) 1.10 (s, 12H); ^{13}C NMR (75 MHz, CD_2Cl_2, 298 K, ppm) δ 149.62, 149.43, 145.58, 140.92, 139.74, 139.38, 132.62, 132.22, 130.48, 129.91, 126.79, 124.83, 124.48, 83.90, 34.67, 34.64, 31.54, 31.45, 24.77; HRMS (MALDI) *m/z*: Calcd for $C_{32}H_{41}BO_2$: 468.3200; Found: 468.3186 $[M]^+$.

Synthesis of [6'-bromo-4,4''-di-*tert*-butyl-(1,1':2',1''-terphenyl)-3'-yl]trimethylsilane (16)

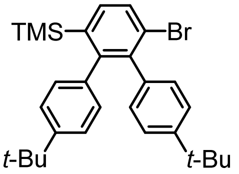

Compound **3** (2.00 g, 4.00 mmol) in 30 mL of anhydrous tetrahydrofuran at −78 °C was added *n*-BuLi (2.5 mL, 1.6 M in hexanes) dropwise. The yellow solution was stirred at −78 °C for 1 h. Trimethylsilyl chloride (521 mg, 4.80 mmol) was added dropwise into the solution. The resulting mixture was gradually warmed to room temperature and kept stirring for 18 h. The reaction mixture was then quenched by adding 20 mL of water. The aqueous phase was extracted by dichloromethane (three times). The combined organic phases were washed with water and dried over magnesium sulfate. After the removal of the solvent under reduced pressure, the residue was purified by column chromatography over silica gel using *n*-hexane as eluent to afford the title compound as a white solid (1.19 g, 60%). M.p.: 146.6 – 147.4 °C. ^{1}H NMR (300 MHz, CD_2Cl_2, 298 K, ppm) δ 7.66 (d, J = 8.1 Hz, 1H), 7.46 – 7.34 (d, J = 8.1 Hz, 1H), 7.14 – 7.07 (m, 4H), 6.85 (td, J = 8.3, 1.9 Hz, 4H), 1.21 (s, 9H), 1.20 (s, 9H), 0.07 (s, 9H); ^{13}C NMR (75 MHz, CD_2Cl_2, 298 K, ppm) δ 150.63, 150.06, 149.68, 142.37, 139.27, 139.15,

138.13, 134.97, 130.93, 130.65, 130.23, 125.50, 124.31, 123.93, 34.63, 34.61, 31.40, 31.38; HRMS (MALDI) *m/z*: Calcd for $C_{29}H_{37}BrSi$: 492.1848; Found: 492.1859 $[M]^+$.

Synthesis of [4,4''-di-*tert*-butyl-6'-(4,4,5,5-tetramethyl-1,3,2-dioxaborolan-2-yl)-(1,1':2',1''-terphenyl)-3'-yl]trimethylsilane (6)

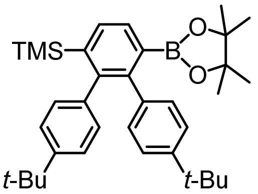

To a solution of compound **15** (1.00 g, 2.03 mmol) in 20 mL of anhydrous tetrahydrofuran was added 0.9 mL of *n*-BuLi (2.5 M in hexanes) dropwise at −78 °C. The yellow solution was stirred at −78 °C for 1 h. 2-Isopropoxy-4,4,5,5-tetramethyl-1,3,2-dioxaborolane (1.13 g, 6.08 mmol) in 2 mL of anhydrous tetrahydrofuran was added dropwise into the solution. The resulting mixture was gradually warmed to room temperature and kept stirring for 12 h. Then, the reaction mixture was quenched by adding 30 mL of water. The aqueous phase was extracted by diethyl ether (three times). The combined organic phases were washed with water and dried over magnesium sulfate. After the removal of the solvent under reduced pressure, the residue was purified by column chromatography over silica gel using *n*-hexane/dichloromethane (5:1 to 3:1) as eluent to afford the title compound as a white solid (825 mg, 75%). M.p.: 165.1 – 166.2 °C. ^{1}H NMR (300 MHz, CD_2Cl_2, 298 K, ppm) δ 7.56 (dd, J = 14.1, 7.4 Hz, 2H), 7.12 (dd, J = 18.0, 8.3 Hz, 4H), 6.89 (dd, J = 15.9, 8.3 Hz, 4H), 1.25 (s, 9H), 1.23 (s, 9H), 1.05 (s, 12), 0.08 (s, 9H); ^{13}C NMR (75 MHz, CD_2Cl_2, 298 K, ppm) δ 146.76, 148.90, 147.29, 145.96, 141.78, 140.14, 139.80, 132.83, 131.52, 131.09, 130.40, 124.02, 123.94, 83.78, 34.64, 34.53, 31.47, 24.71, 0.57; HRMS (MALDI) *m/z*: Calcd for $C_{35}H_{49}BO_2Si$: 540.3595; Found: 540.3624 $[M]^+$.

Synthesis of 2-[6'-bromo-4,4''-di-*tert*-butyl-(1,1':2',1''-terphenyl)-3'-yl]-4,4,5,5-tetramethyl-1,3,2-dioxaborolane (10)

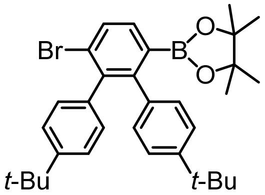

To a solution of compound **3** (2.38 g, 4.76 mmol) in 50 mL of anhydrous THF was added 2.1 mL of *n*-BuLi (2.5 M in *n*-hexane) dropwise at −78 °C. The yellow solution was stirred at −78 °C for 1 h. 2-Isopropoxy-4,4,5,5-tetramethyl-1,3,2-dioxaborolane (1.77 g, 9.52 mmol) in 3.5 mL of anhydrous THF was added dropwise into the solution. The resulting mixture was gradually warmed to room temperature and kept stirring for 12 h followed by quenching with 30 mL of water. The aqueous phase was extracted by diethyl ether (three times). The combined organic phases were washed with water and dried over magnesium sulfate. After the removal of the solvent under reduced pressure, the residue was purified by column chromatography over silica gel using *n*-hexane/dichloromethane (2:1) as eluent to afford the title compound as a white solid (1.92 g, 74%). M.p.: 225.1 – 225.7 °C. ^{1}H NMR (300 MHz, CD_2Cl_2, 298 K, ppm) δ 7.65 (d, J = 8.0 Hz, 1H), 7.44 (d, J = 8.0 Hz, 1H), 7.19 (d, J = 8.2 Hz, 2H), 7.09 (d, J = 8.1 Hz, 2H), 6.92 (d, J = 8.2 Hz, 2H), 6.84 (d, J = 8.2 Hz, 2H), 1.25 (s, 9H), 1.22 (s, 9H), 1.06 (s, 12H); ^{13}C NMR (75 MHz, CD_2Cl_2, 298 K, ppm) δ 150.03, 149.60, 148.89, 141.83, 139.18, 137.83, 133.85,

131.09, 130.41, 130.08, 126.69, 124.50, 124.09, 84.06, 34.70, 34.57, 31.42, 31.40, 24.69; HRMS (MALDI) *m/z*: Calcd for $C_{32}H_{40}BBrO_2$: 546.2305; Found: 546.2317 $[M]^+$.

Synthesis of compound 5

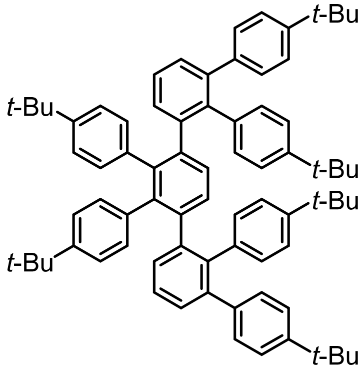

Compound **3** (1.20 g, 2.56 mmol), compound **4** (512 mg, 1.02 mmol), tripotassium phosphate (1.09 g, 5.12 mmol) and [1,1'-bis(diphenylphosphino)ferrocene] dichloropalladium(II) (74.9 mg, 0.102 mmol) were dissolved in a mixture of dimethylformamide (20 mL) and water (2 mL) followed by degassing via bubbling through argon for 0.5 h. The resulting mixture was heated to 90 °C and stirred for 19 h. After cooling to room temperature, 50 mL of water was added. The aqueous phase was extracted by dichloromethane (three times). The combined organic phases were washed with water and dried over magnesium sulfate. After the removal of the solvent under reduced pressure, the residue was purified by column chromatography over silica gel using *n*-hexane/dichloromethane (5:1) as eluent to afford the title compound as a white solid (750 mg, 72%). M.p: > 300 °C. ^{1}H NMR (500 MHz, $C_2D_2Cl_4$, 393 K, ppm) δ 7.26 (t, J = 4.5 Hz, 2H), 7.19 (d, J = 4.8 Hz, 4H), 7.11 (m, 6H), 6.97 (d, J = 7.8 Hz, 4H), 6.90 (d, J = 7.9 Hz, 4H), 6.73 (d, J = 8.0 Hz, 4H), 6.51 (d, J = 7.8 Hz, 4H), 6.30 (d, J = 7.9 Hz, 4H), 1.33 (s, 18H), 1.30 (s, 18H), 1.18 (s, 18H); ^{13}C NMR (125 MHz, $C_2D_2Cl_4$, 393 K, ppm) δ 148.44, 148.10, 147.42, 141.70, 141.50, 140.32, 139.74, 139.41, 139.38, 136.68, 136.28, 131.42, 131.22, 130.75, 129.42, 129.36, 128.22, 125.35, 123.65, 122.84, 122.40, 33.91, 33.67, 31.14, 31.11, 31.05, 30.97; HRMS (MALDI) *m/z*: Calcd for $C_{78}H_{86}$: 1022.6730; Found: 1022.6765 $[M]^+$.

Synthesis of compound 7

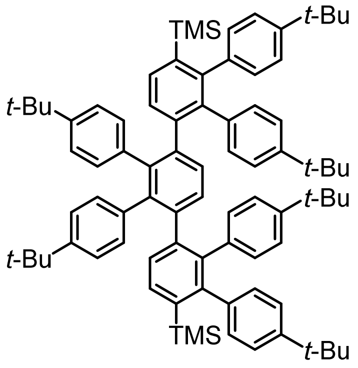

To a mixture of compound **3** (293 mg, 0.586 mmol.), compound **6** (700 mg, 1.29 mmol), and tetrakis (triphenylphosphine)palladium(0) (74.9 mg, 64.8 µmol) was added 1.8 mL of aqueous potassium carbonate solution (2.0 M) and 20 mL of 1,4-dioxane. The mixture was degassed via bubbling through

argon for 0.5 h. Then, the resulting mixture was heated to 90 °C and stirred for 12 h. After cooling to room temperature, 50 mL of water was added. The aqueous phase was extracted by dichloromethane (three times). The combined organic phases were washed with water and dried over magnesium sulfate. After the removal of the solvent under reduced pressure, the residue was purified by column chromatography over silica gel using *n*-hexane/dichloromethane (5:1) as eluent to afford the title compound as a white solid (564 mg, 82%). M.p.: 138.6 – 139.2 °C. ^{1}H NMR (500 MHz, $C_2D_2Cl_4$, 393 K, ppm) δ 7.37 (d, J = 7.8 Hz, 2H), 7.12 (d, J = 8.1 Hz, 4H), 7.03 (d, J = 8 Hz, 4H), 6.84 (d, J = 8.0 Hz, 4H), 6.77 (d, J = 8.1 Hz, 8H), 6.39 (d, J = 7.7 Hz, 4H), 6.35 (d, J = 7.7 Hz, 4H), 1.27 (s, 18H), 1.26 (s, 18H), 1.22 (s, 18H), 0.03 (s, 18H); ^{13}C NMR (125 MHz, $C_2D_2Cl_4$, 393 K, ppm) δ 148.78, 147.77, 147.45, 147.37, 141.74, 140.01, 139.89, 139.74, 136.85, 136.41, 131.52, 131.47, 131.26, 130.74, 129.81, 129.14, 122.81, 122.33, 122.30, 33.89, 33.78, 33.71, 31.10, 31.07, 31.01, 0.40; HRMS (MALDI) *m/z*: Calcd for $C_{84}H_{102}Si_2$: 1166.7520; Found: 1166.7558 [M]$^+$.

Synthesis of compound 8

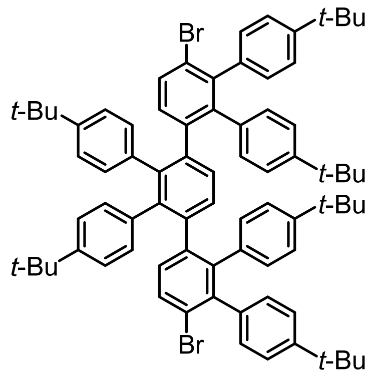

To a solution of compound **7** (308 mg, 0.264 mmol) in 10 mL of methanol and 10 mL of dichloromethane was added bromine (132 mg, 0.826 mmol) dropwise. The mixture was stirred at room temperature for 12 h. The reaction was then quenched by saturated aqueous sodium sulfite solution. The aqueous phase was extracted by dichloromethane (three times). The combined organic phases were washed with water and dried over magnesium sulfate. After the removal of the solvent under reduced pressure, the residue was purified by column chromatography over silica gel using *n*-hexane/dichloromethane (5:1) as eluent to afford the title compound as a white solid (306 mg, 98%). M.p.: 119.6 – 120.2 °C. ^{1}H NMR (500 MHz, $C_2D_2Cl_4$, 393 K, ppm) δ 7.48 (d, J = 8.6 Hz, 2H), 7.15 – 7.02 (m, 8H), 6.86 (d, J = 8.1 Hz, 4H), 6.83 – 6.77 (m, 8H), 6.37 (d, J = 8.0 Hz, 4H), 6.28 (d, J = 8.2 Hz, 4H), 1.28 (s, 18H), 1.27 (s, 18H), 1.22 (s, 18H); ^{13}C NMR (125 MHz, $C_2D_2Cl_4$, 393 K, ppm) δ 149.10, 148.08, 147.70, 142.55, 141.87, 140.87, 140.25, 138.91, 137.68, 136.18, 135.72, 132.16, 131.34, 130.99, 130.49, 130.21, 129.92, 123.91, 123.68, 122.90, 122.32, 34.28, 34.14, 34.03, 32.00, 31.38, 31.33, 31.28, 29.80, 29.75, 29.45, 22.81, 14.34; HRMS (MALDI) *m/z*: Calcd for $C_{78}H_{84}Br_2$: 1178.4940; Found: 1178.5010 [M]$^+$.

Synthesis of compound 9

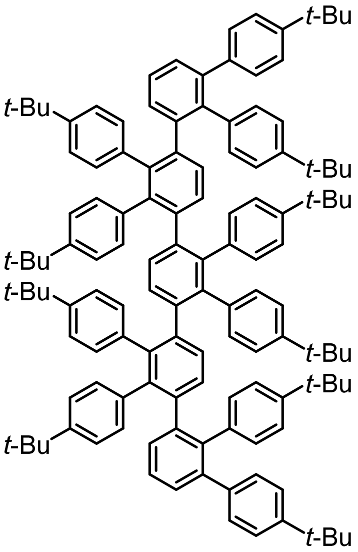

Compound **8** (28.4 mg, 24.0 µmol), compound **4** (33.8 mg, 72.1 µmol), tripotassium phosphate (25.5 mg, 120 µmol) and [1,1'-bis(diphenylphosphino)ferrocene] dichloropalladium(II) (1.80 mg, 2.40 µmol) were dissolved in a mixture of dimethylformamide (5 mL) and water (0.5 mL). The mixture was degassed via bubbling through argon for 0.5 h. The resulting mixture was heated to 90 ℃ and stirred for 24 h. After cooling to room temperature, 20 mL of water was added. The aqueous phase was extracted by dichloromethane (three times). The combined organic phases were washed with water and dried over magnesium sulfate. After the removal of the solvent under reduced pressure, the residue was purified by column chromatography over silica gel using *n*-hexane/dichloromethane (5:1) as eluent to afford the title compound as a white solid (28.7 mg, 70%). M.p: > 300 °C. ^{1}H NMR (500 MHz, $C_2D_2Cl_4$, 393 K, ppm) δ 7.27 – 6.68 (m, 37H), 6.47 (d, J = 8.1 Hz, 4H), 6.32 – 6.21 (m, 11H) 1.34 – 1.25 (m, 72H), 1.19 (s, 18H); ^{13}C NMR (125 MHz, $C_2D_2Cl_4$, 393 K, ppm) δ 148.28, 147.87, 147.18, 147.11, 147.00, 141.68, 141.02, 140.00, 139.86, 139.62, 139.28, 139.13, 139.08, 139.04, 136.58, 136.50, 135.91, 131.41, 130.77, 129.67, 129.59, 129.37, 129.29, 128.32, 125.63, 125.49, 123.95, 123.88, 123.12, 122.41, 34.04, 33.89, 33.78, 31.27, 31.22, 31.17, 31.14, 31.10; HRMS (MALDI) *m/z*: Calcd for $C_{130}H_{142}$: 1703.1112; Found: 1703.1168 $[M]^+$.

Synthesis of triphenanthro-fused teropyrene 1

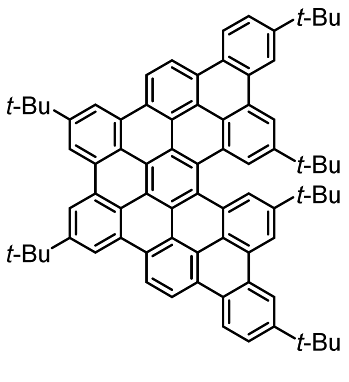

A solution of compound **5** (20.0 mg, 19.5 µmol) in 30 mL of unstabilized dichloromethane was degassed by argon bubbling for 10 min. To the degassed solution was added a suspension of iron(III) chloride (285 mg, 1.76 mmol) in 1.5 mL of nitromethane. After stirring at room temperature for 5 h

under continuous bubbling with argon pre-saturated unstabilized dichloromethane, the reaction was quenched with 5 mL of methanol. Then, 20 mL of water was added followed by extracting with dichloromethane (three times). The combined organic phases were washed with water and dried over magnesium sulfate. After the removal of the solvent under reduced pressure, the residue was purified by column chromatography over silica gel using *n*-hexane/dichloromethane (2:1) as eluent to afford **1** as a yellowish-green solid (15.6 mg, 79%). M.p.: > 300 °C. ^{1}H NMR (500 MHz, CD_2Cl_2, 298 K, ppm) δ 9.54 (d, J = 8.8 Hz, 2H), 9.39 (s, 2H), 9.33 (d, J = 8.7 Hz, 4H), 9.01 (s, 2H), 8.99 (d, J = 8.7 Hz, 2H), 8.93 (s, 2H), 8.89 (s, 2H), 7.97 (dd, J = 8.5, 1.9 Hz, 2H) 1.84 (s, 18H), 1.66 (s, 18H), 1.27 (s, 18H); ^{13}C NMR (125 MHz, CD_2Cl_2, 298 K, ppm) δ 151.24, 150.35, 148.21, 131.24, 131,12, 130.84, 130.30, 128.77, 128.61, 128.57, 128.16, 126.31, 124.81, 124.71, 124.66, 124.63, 124.38, 124.11, 124.08, 122.17, 122.05, 121.73, 120.16, 120.06, 119.69, 119.02, 36.37, 35.76, 35.49, 32.33, 31.84, 31.77; HRMS (MALDI) *m/z*: Calcd for $C_{78}H_{72}$: 1008.5634; Found: 1008.5622 $[M]^{+}$.

Synthesis of pentaphenanthro-fused quateropyrene 2

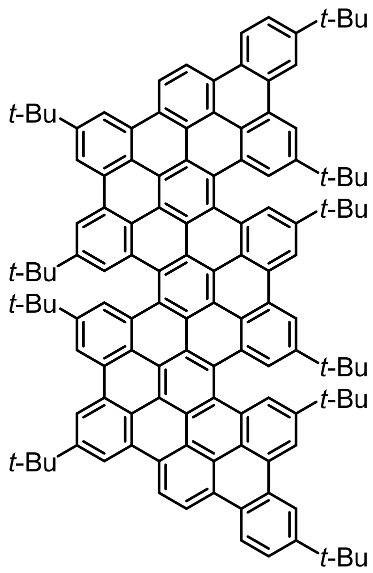

A solution of compound **9** (50.0 mg, 29.3 μmol) in 35 mL of unstabilized dichloromethane was degassed by argon bubbling for 0.5 h. To the degassed solution was added a pre-degassed suspension of iron(III) chloride (693 mg, 1.12 mmol) in 6.0 mL of nitromethane. After stirring at 45 °C for 3 h, the reaction was quenched by addition of 7 mL of methanol. Then, 20 mL of water was added followed by extracting with dichloromethane (three times). The combined organic phases were washed with water and dried over magnesium sulfate. After the removal of the solvent under reduced pressure, the residue was purified by column chromatography over silica gel using *n*-hexane/dichloromethane (5:1) as eluent to afford **2** as a red solid (26.4 mg, 54% yield). M.p.: > 300 °C. ^{1}H NMR (500 MHz, $C_2D_2Cl_4$, 393 K, ppm) δ 9.67 – 8.97 (m, 24H), 8.05 (d, J = 8.5 Hz, 2H), 1.98 (s, 18H), 1.77 (s, 18H), 1.55 – 1.40 (m, 54H); ^{13}C NMR (125 MHz, $C_2D_2Cl_4$, 393 K, ppm) δ 150.65, 149.92, 148.06, 131.13, 131.10, 130.63, 130.57, 130.55, 128.16, 127.98, 127.77, 125.32, 124.85, 124.33, 124.07, 123.73, 123.67, 123.63, 121.40, 121.36, 121.29, 121.10, 119.69, 119.60, 119.33, 119.07, 119.00, 118.94, 118.89, 118.80, 118.44, 74.00, 73.78, 73.56, 35.00, 31.88, 31.51, 31.48, 31.45, 31.42, 31.39, 31.34, 31.32, 31.29, 30.85; HRMS (MALDI) *m/z*: Calcd for $C_{130}H_{116}$: 1676.9077; Found: 1676.9041 $[M]^{+}$.

Synthesis of P1

To a Schlenk tube was added compound **10** (300 mg, 0.548 mmol), bis(tri-*tert*-butylphosphine)palladium(0) (7.00 mg, 13.7 μmol), 1.4 mL of degassed aqueous tripotassium phosphate solution (3 M), and 1.4 mL of degassed THF under argon. The mixture was stirred at 50 °C for 24 h. Then, bromobenzene (86.0 mg, 0.548 mmol) was added, and the resulting mixture was stirred for 12 h. Subsequently, phenyboronic acid (66.8 mg, 0.548 mmol) in 0.5 mL of degassed THF was added, and the resulting mixture was stirred for another 12 h. Then, 20 mL of water was added before the aqueous phase was extracted by dichloromethane (three times). The combined organic phases were washed with water and dried over magnesium sulfate. After the removal of the solvent under reduced pressure, the residue was dissolved in 5 mL of dichloromethane followed by precipitation into 100 mL of methanol. The white precipitates were collected via filtration and dried under vacuum. The white solid was further washed by Soxhlet extraction with boiling acetone for 2 days, and then dried under reduced pressure at room temperature to afford **P1** as a white solid (184 mg, 98%). FT-IR (powder) 3085, 3052, 3033, 2958, 2902, 2866, 1514, 1477, 1461, 1440, 1391, 1361, 1268, 1199, 1109, 1028, 1006, 942, 828, 780, 689, 655, 640, 581 cm^{-1}.

Synthesis of FGNR

A solution of **P1** (20.0 mg) in 25 mL of unstabilized dichloromethane was degassed by argon bubbling for 20 min. To the degassed solution was added dropwise a degassed suspension of iron(III) chloride (324 mg) in 3 mL of nitromethane. After stirring at 45 °C for 48 h, the reaction was quenched by addition of 10 mL of methanol. The precipitates were collected via filtration. Then, the collected solid was re-dispersed in 10 mL of THF followed by adding dropwise into 100 mL of methanol. The precipitates were collected via filtration followed by washing intensively with methanol and dried under vacuum to afford **FGNR** as a dark red solid (17.4 mg, 88% yield). FT-IR (powder) 2958, 2924, 2866, 1702, 1598, 1461, 1397, 1365, 1341, 1249, 1200, 1123, 1073, 929, 899, 869, 838, 752, 658, 587 cm^{-1}.

3. Mass spectra

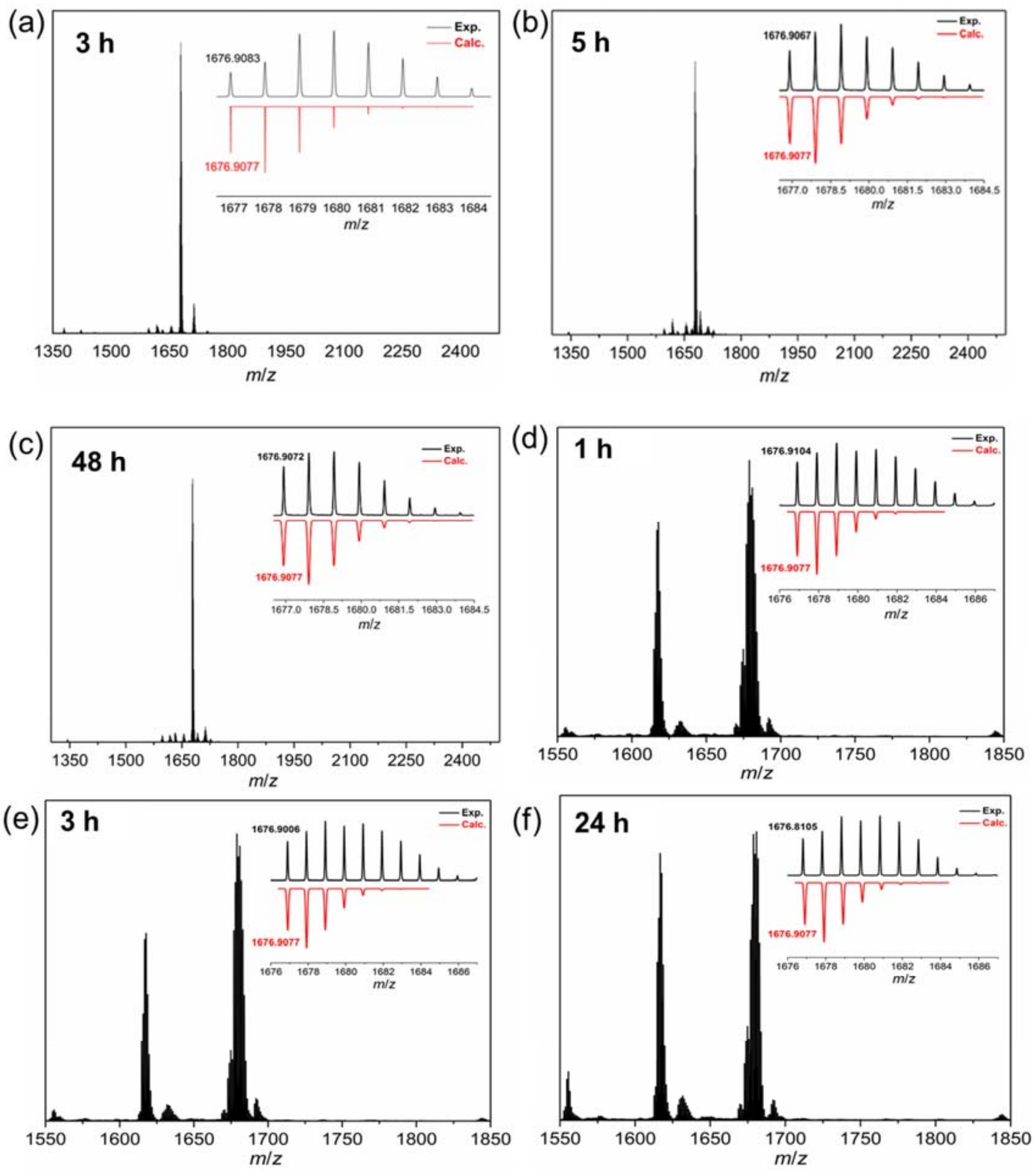

Figure S1. MALDI-TOF mass spectrometry (MS) results of cyclodehydrogenation of **9** toward **2** under different conditions: (a-c) iron(III) chloride; (d-f) 2,3-dichloro-5,6-dicyano-1,4-benzoquinone (DDQ)/ trifluoromethanesulfonic acid (CF_3SO_3H).

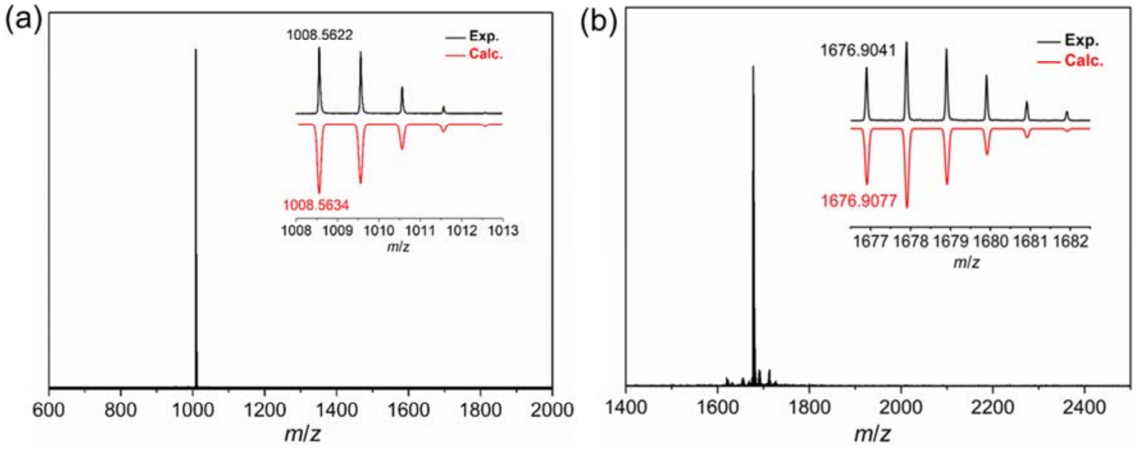

Figure S2. High-resolution MALDI-TOF MS results of (a) **1** and (b) **2**; inset: the corresponding experimental and simulated isotopic distributions.

4. Isomerization processes of 1 and 2

To evaluate the isomerization processes of **1** and **2**, DFT calculations were performed. As illustrated in Figure S3, the interconversion between [*M*]- and [*P*]-**1** was proposed to adopt a transition state where terminal benzene rings of the [5]helicene substructure are oriented face-to-face because of the bulky *tert*-butyl groups (Figure S3a). Accordingly, the isomerization barrier was estimated to be 36.0 kcal/mol which is by 13 kcal/mol higher than that of [5]helicene.[2] In addition to [*M*,*M*,*M*]- and [*P*,*P*,*P*]-**2** observed in the X-ray crystallography of **2**, presence of their diastereomers was expected considering the up-down fluctuation of π-surfaces resulting from [5]helicene moieties. To catch a glimpse of the isomerization pathway of **2**, computation of a specific interconversion between [*M*,*M*,*M*]-**2** and [*P*,*P*,*P*]-**2** was performed. As demonstrated in Figure S3b, there are two plausible pathways, involving five transition states and three metastable diastereomers ([*M*,*P*,*M*]-, [*M*,*M*,*P*]- and [*M*,*P*,*P*]-**2**). The isomerization barrier estimated for each transition step surpassed 36 kcal/mol, ensuring stability against the interconversion at room temperature.

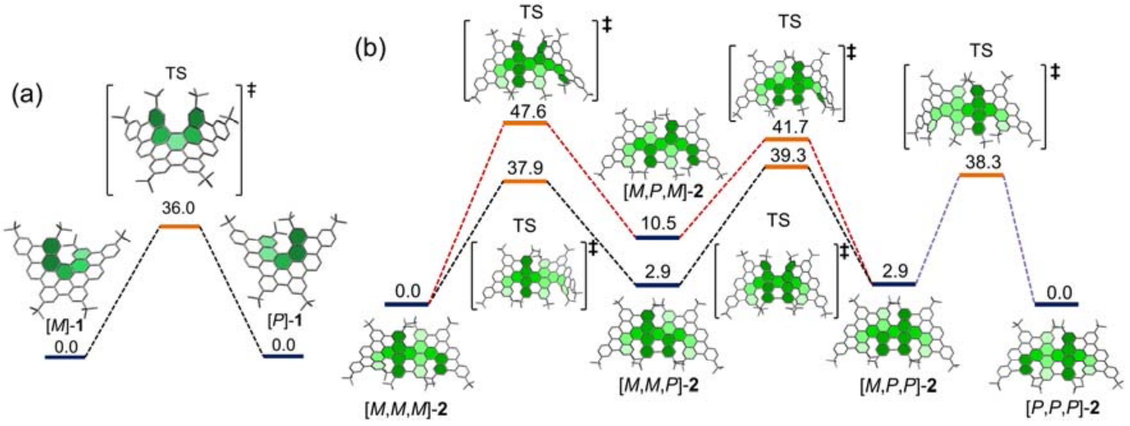

Figure S3. (a) Isomerization process of **1** from [*M*]-**1** to [*P*]-**1**. (b) Isomerization processes of **2** from [*M*,*M*,*M*]-**2** to [*P*,*P*,*P*]-**2** based on its crystal structure. Two different isomerization processes are proposed for **2**. The relative Gibbs free energy Δ*G* (kcal/mol) was calculated at the B3LYP/6-31G(d,p) level. TS: transition state. The sequence of helicity annotated in **2** is from left to right.

5. Optical resolution by HPLC

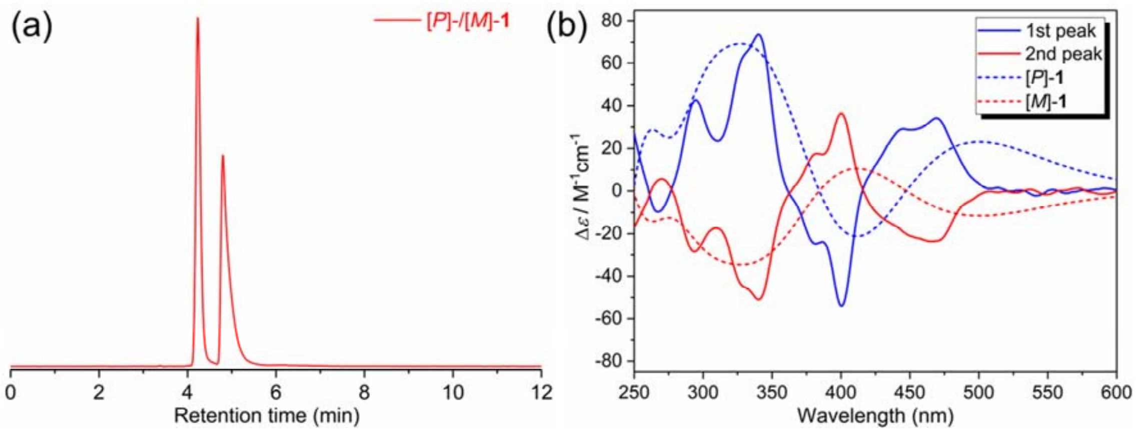

Figure S4. (a) Chiral HPLC traces during the separation of **1** monitored at 400 nm. A mixture of isopropanol/*n*-hexane = 1:9 was used as the eluent with a flow rate of 1 mL/min. (b) CD spectra of the two enantiomers of **1** separated by chiral HPLC in CH_2Cl_2 and simulated CD spectra of [*P*]- and [*M*]-**1** based on time-dependent density functional theory (TD-DFT) calculations at the B3LYP/6-31G(d, p) level.

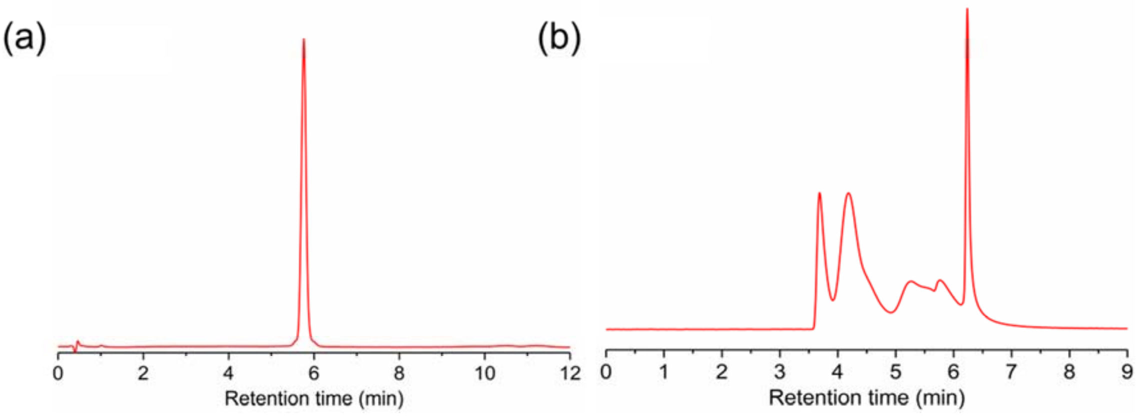

Figure S5. (a) HPLC traces (THF/H_2O, gradient condition: from 60%:40% (0 min) to 100%:0% (10 min)) of **2** monitored at 260 nm with a flow rate of 1 mL/min. (b) Chiral HPLC traces of **2** monitored at 425 nm. A mixture of isopropanol/*n*-hexane = 2:98 was used as the eluent with a flow rate of 1 mL/min. Enantiomeric resolution of **2** with chiral HPLC exhibited five distinct bands, indicating a mixture of different enantiomers.

6. SEC and MALDI-TOF MS analysis of P1

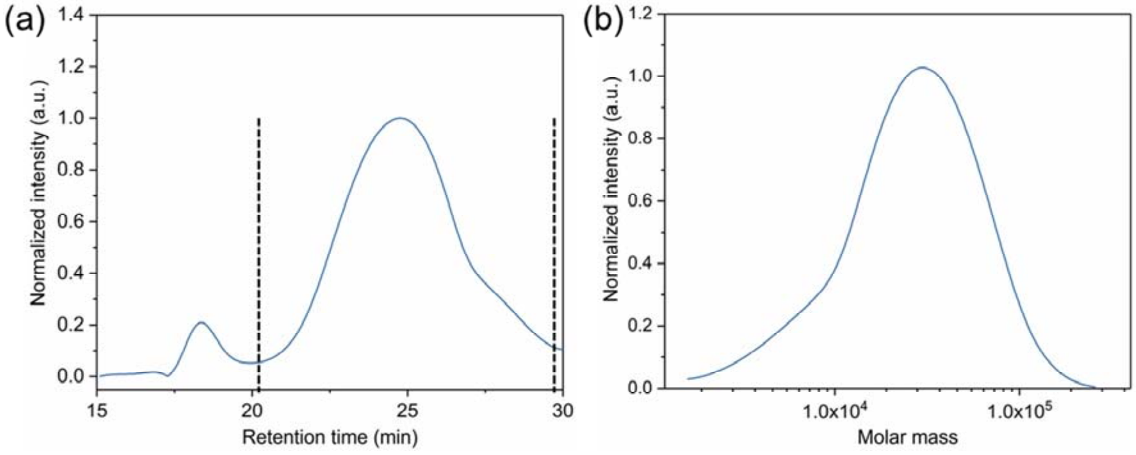

Figure S6. SEC characterization of **P1**. (a) SEC chart of **P1** after Soxhlet extraction with acetone. (b) Molecular weight distribution calculated from the selected region in (a) between the dashed lines.

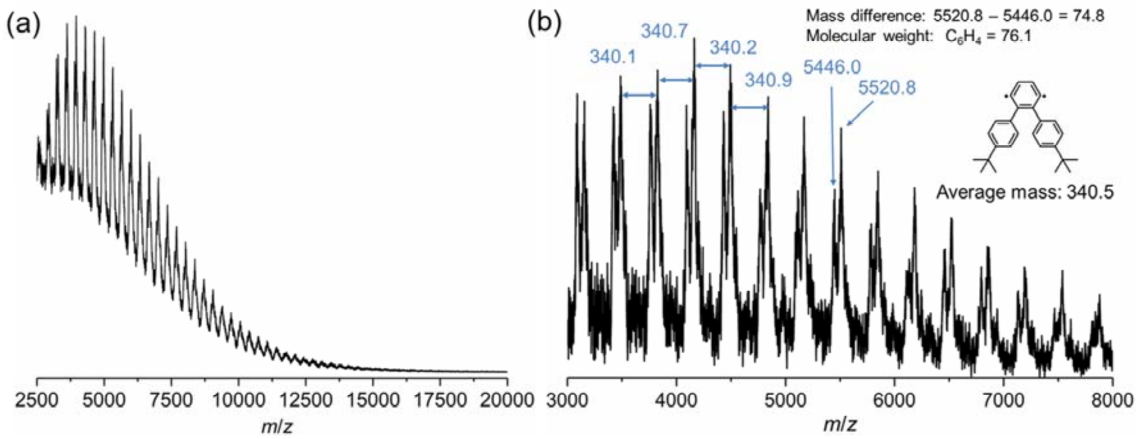

Figure S7. MALDI-TOF spectrum of **P1** (matrix: tetracyanoquinodimethane, linear mode). (a) Full range spectrum. (b) Zoom in spectrum (baseline was subtracted). The inset shows the chemical structure and average mass of the repeating unit of **P1**. Two sets of peaks were observed for each oligomer, corresponding to those with and without the phenyl end group.

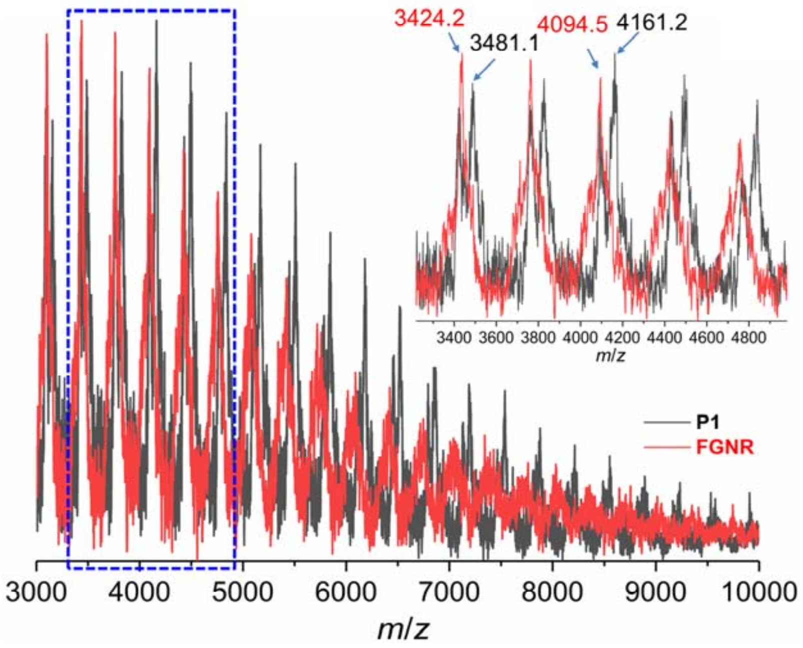

Figure S8. The comparison of MALDI-TOF spectra of **P1** and **FGNR** (matrix: tetracyanoquinodimethane, linear mode). Inset: magnified region indicated by blue dashed frame showing the mass difference between **P1** and **FGNR**. The representing number indicates the mass of **P1** and **FGNR** with 10 and 12 repeating units terminated with one phenyl end-group.

The solvent-free sample preparation allows **FGNR** to be detected as radical cation under the MALDI condition, similar to polycyclic aromatic hydrocarbons (PAHs) and fullerenes.[3-5] The observed mass result of **FGNR** was used directly to evaluate cyclodehydrogenation efficiency by comparing the number of hydrogen atoms lost during the Scholl reaction, derived from mass difference between corresponding peaks of **P1** and **FGNR** in their MALDI-TOF MS, with the theoretical number of hydrogen atoms removed.[6]

Table S1. Estimation of the cyclodehydrogenation efficiency based on MALDI-TOF MS.

[a]n	9	10	11	12	13	14	15
[b]$N(H_{calc.})$	52	56	64	70	76	82	88
[c]$N(H_{exp.})$	52	56	62	70	72	78	82
$N(H_{exp}.)/N(H_{calc.})$	1.00	1.00	0.97	1.00	0.95	0.95	0.93

[a]n: Number of repeating unit.

[b]$N(H_{calc.})$: Calculated number of hydrogen atoms removed during the cyclodehydrogenation.

[c]$N(H_{exp.})$: Number of lost hydrogen atoms derived from MALDI-TOF results of **P1** and **FGNR**.

7. FT-IR spectrum

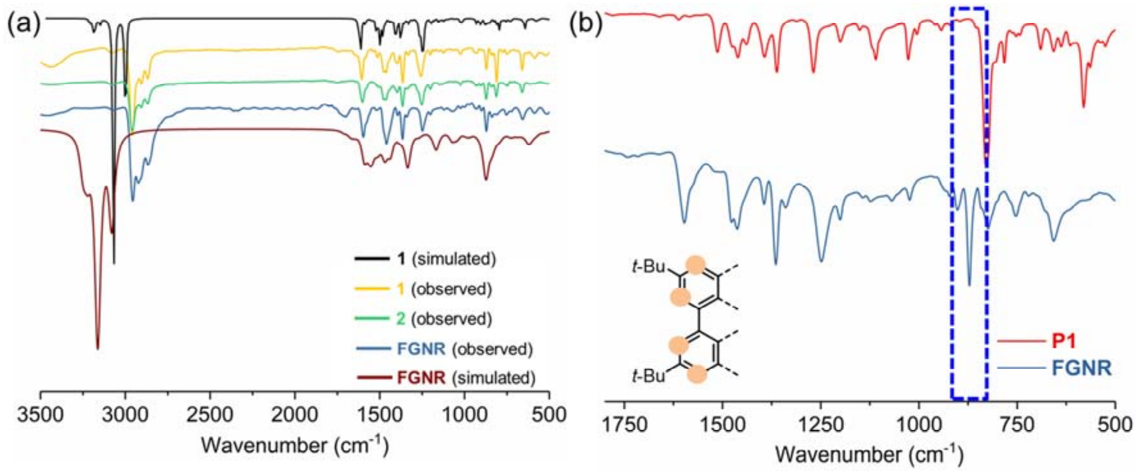

Figure S9. (a) Full FT-IR spectra of **1**, **2** and **FGNR** as well as simulated spectra of **1** and **FGNR**. (b) FT-IR spectra of **P1** and **FGNR**. Blue dashed rectangle indicates a peak from SOLO mode at 870 cm^{-1} (wagging of an isolated aromatic C-H bond neighbored by two C-C bonds, orange-colored in inset of Figure S9b), which is absent in the spectrum of **P1**.

8. Electrochemical properties of 1 and 2

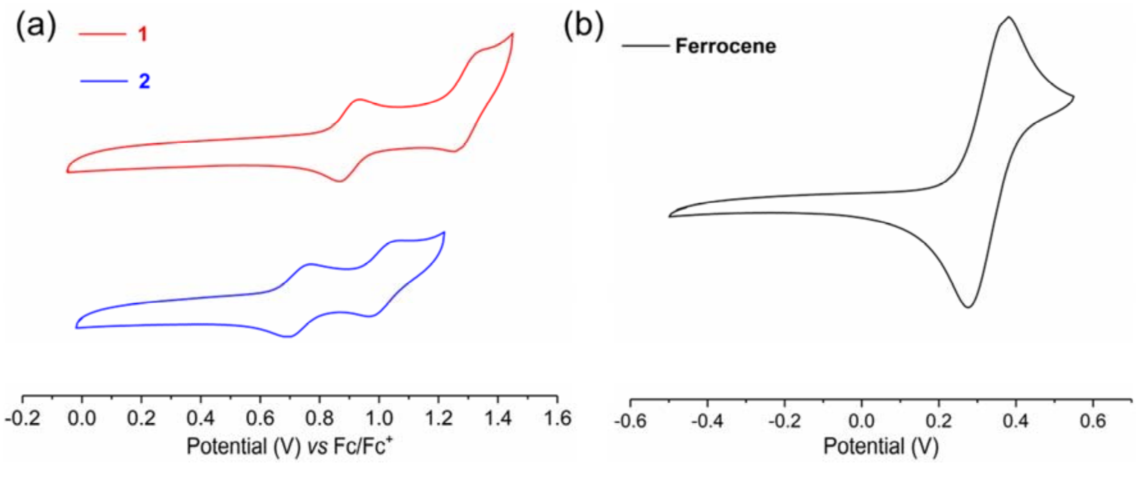

Figure S10. Cyclic voltammograms of (a) **1** and **2**; (b) ferrocene. Only the oxidation process was observed for both **1** and **2**. HOMO was estimated with ferrocene as an external standard. The oxidation potential of ferrocene was regarded as −4.8 eV from the vacuum level.

Table S2. Summary of the photophysical and electrochemical properties of **1** and **2**.

Compound	λ_{max}^{abs}/nm	λ_{max}^{em}/nm	$^{a}E_{g}^{opt}$/eV	^{b}HOMO/eV	^{c}HOMO/eV
**1**	398	502	2.52	–5.37	−4.72
**2**	427	561	2.25	–5.19	−4.56

[a] Optical energy gap deduced from the absorption onset.

[b] HOMO energy level obtained from cyclic voltammograms

[c] HOMO energy level obtained from DFT calculation at the HSE06/6-31G(d) level.

9. X-ray crystallographic analysis of 1 and 2

The X-ray crystallographic coordinates for structures reported in this work have been deposited at the Cambridge Crystallographic Data Centre (CCDC), under deposition number 2058017 (**1**) and 2058018 (**2**). These data can be obtained free of charge from CCDC via http://www.ccdc.cam.ac.uk/data_request/cif.

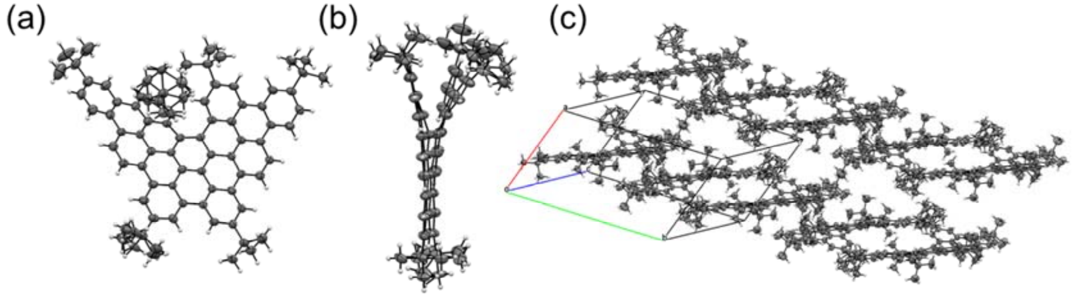

Figure S11. X-ray single-crystal structure of **1** with thermal ellipsoids set at 50% probability level. (a) front view; (b) side view and (c) three-dimensional packing mode.

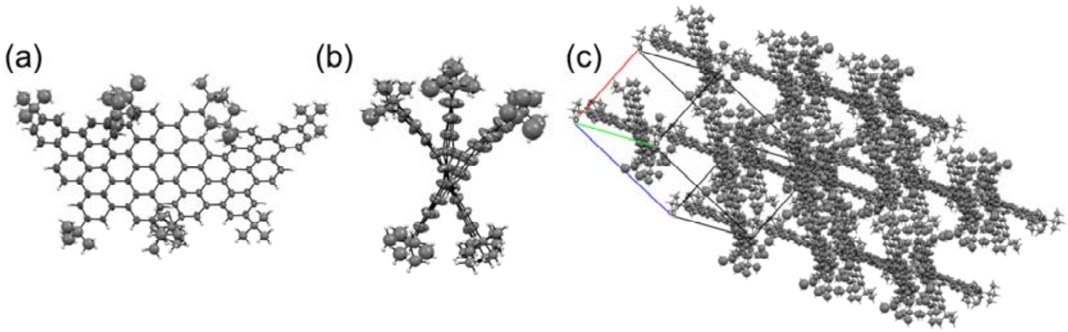

Figure S12. X-ray single-crystal structure of **2** with thermal ellipsoids set at 50% probability level. (a) front view; (b) side view and (c) three-dimensional packing mode.

Helicity of model compound **1** is obvious as only one [5]helicene subunit embedded. However, the situation becomes more complex for model compound **2** with two more incorporated [5]helicene subunits. Both NMR and chiral HPLC spectrum of **2** indicated the existence of multiple conformers, but only [*M*,*M*,*M*]-**2** and [*P*,*P*,*P*]-**2** were found in its crystal structures. In order to elucidate the conformations of compound **2**, we performed the theoretical optimization (DFT-B3LYP/6-31G(d,p)) of the three possible diastereomers, [*M*,*M*,*M*]-, [*M*,*M*,*P*]-, and [*P*,*M*,*P*]-**2**, as shown in in Figure S13. Comparison of the relative stabilities of these three diastereomers of **2** suggested that [*M*,*M*,*M*]- and [*M*,*M*,*P*]-**2** have similar stability, consistent with the experimental observations indicating the existence of multiple diastereomers.

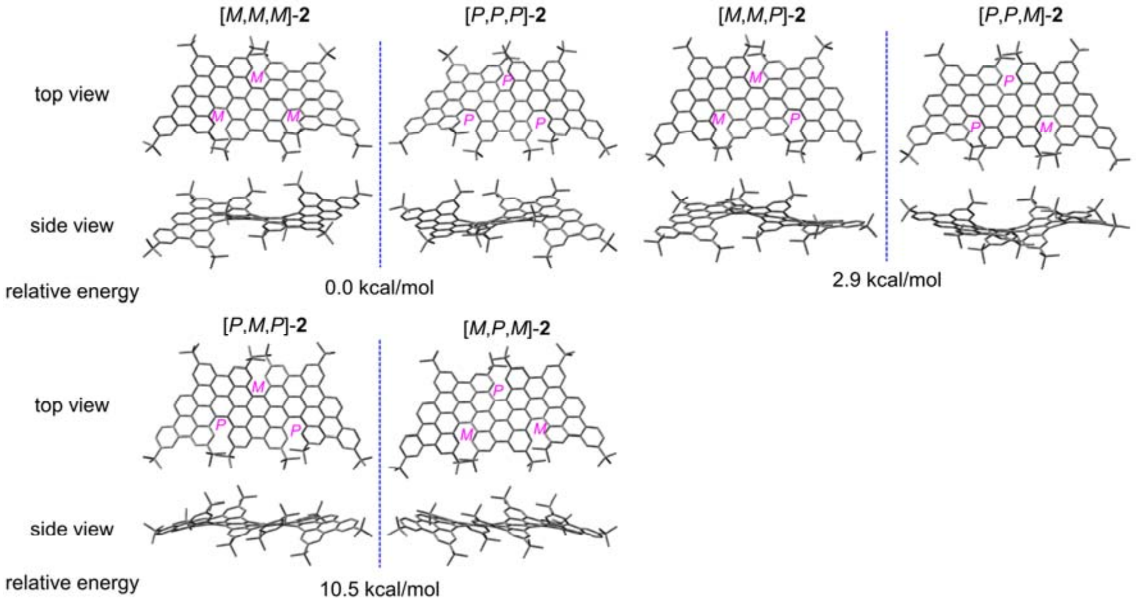

Figure S13. DFT-optimized geometries of **2** calculated at the B3LYP/6-31G(d,p) level. The sequence of helicity annotated in **2** is from left to right.

10. DFT calculations

DFT computations were carried out by using the Gaussian 16 software package.[7] Different short oligomers and infinite GNR (using periodic boundary condition) were computed at the DFT level of theory with the HSE06 functional and 6-31G(d) basis set. Geometry optimization are followed by frequency calculations to obtain the IR spectra and by TD-DFT single point calculations to obtain the absorption spectra. In order to create the repeating unit for the infinite **FGNR**, **FGNR-monomer** (as shown in Figure S14b) is considered. *tert*-Butyl substituent is considered for all the oligomers and **FGNR**.

Geometry of the optimized structures

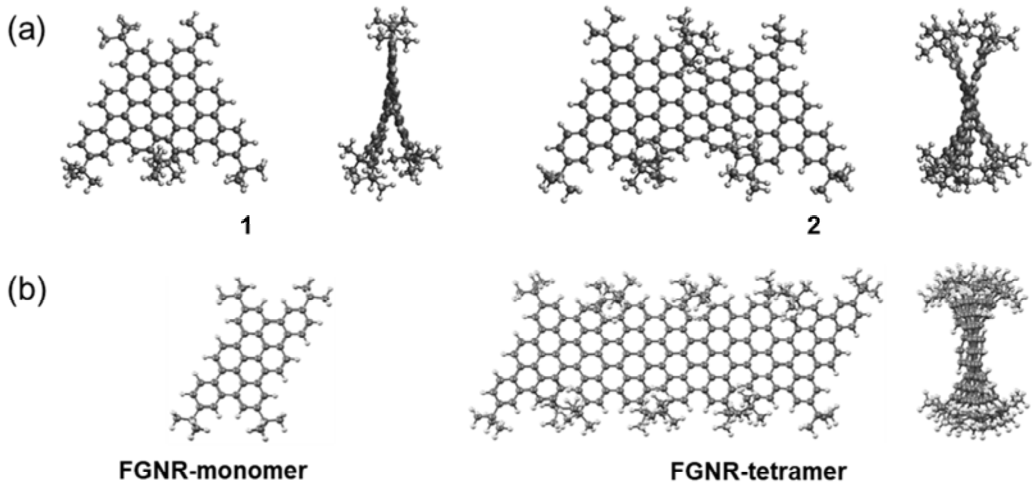

Figure S14. Optimized structures. (a) Model compound **1** and **2**. (b) **FGNR-monomer** and **FGNR-tetramer**.

Electronic properties

The extension of π-conjugation reduces the energy gap. The energy gap decreases from 2.65 to 2.08 eV going from the **1** to **FGNR-tetramer**. The band gap of **FGNR** is similar to the energy gap obtained for **FGNR-tetramer**, with a value of 1.93 eV, suggesting reaching of saturation already for short oligomers, as shown in Figure S15. The optical energy gaps of **1** and **2** deduced from the absorption onset are 2.52 and 2.25 eV, respectively. It should be noted that **1** displays a smaller gap compared to its analog with a flat-lying geometry (2.64 eV),[8] indicating the impact of distortion on electronic property.

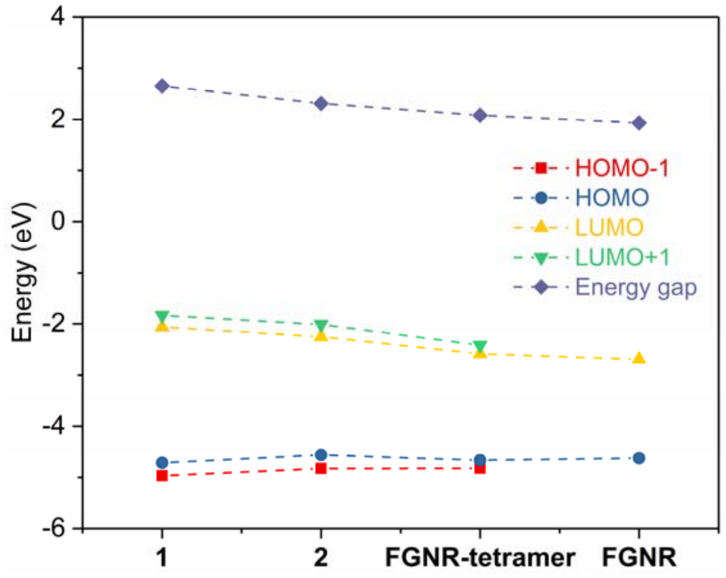

Figure S15. Calculated energy level of **1**, **2**, **FGNR-tetramer**, and infinite **FGNR**.

The shapes of the frontier orbitals of **1**, **2**, and **FGNR-tetramer** are reported in Figure S16. All the HOMO orbitals propagate along the length direction, while the LUMO are oriented more along the width direction.

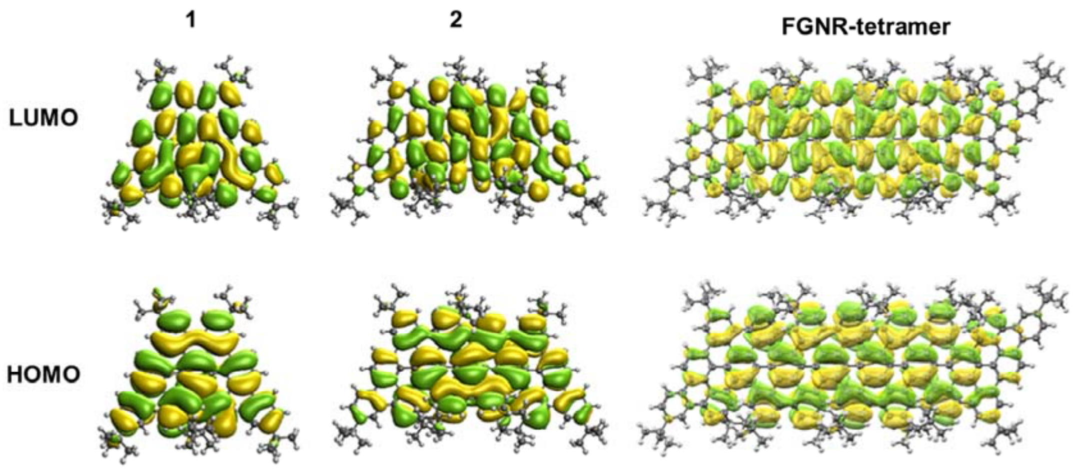

Figure S16. The shapes of the frontier orbitals of **1**, **2**, and **FGNR-tetramer**.

Optical properties

The absorption spectra of **1**, **2**, and **FGNR-tetramer** have been calculated and is reported in Figure S17. A strong red shift is observed going from **1** to **FGNR-tetramer**, with the lowest absorption peak shifting from 466 to 600 nm. In specific, a total shift of 72 nm going from **1** to **2** and of 62 nm from **2** to **FGNR-tetramer** is demonstrated. The longest wavelength absorption is described by a HOMO to LUMO transition for all oligomers.

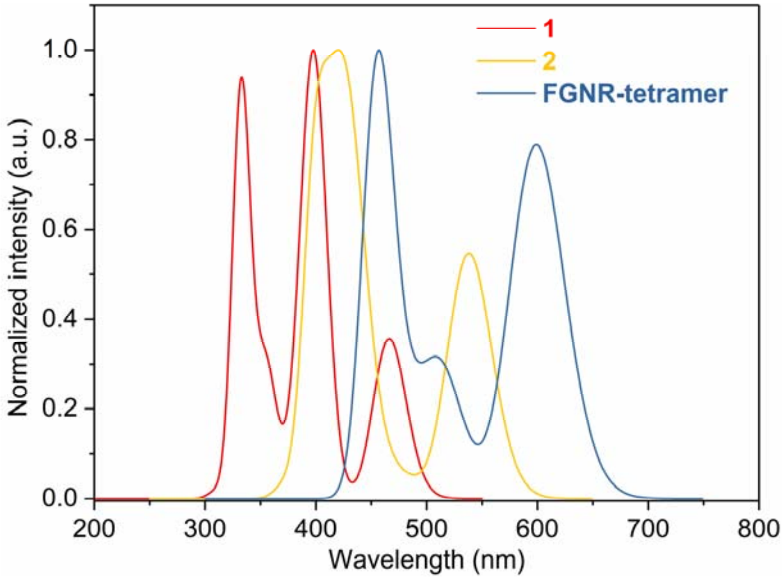

Figure S17. Calculated UV-vis absorption of **1**, **2**, and **FGNR-tetramer**.

The absorption spectra are quite complex and show many peaks. Table S3 summarises the simulated transitions of **1**, **2**, and **FGNR-tetramer**.

Table S3. The transitions at each absorption band of **1**, **2**, and **FGNR-tetramer**.

	**Energy (nm)**	**Oscillator strength (f)**	**Transition**
**1**			
S1	466	0.26	H → L
S3	400	0.62	H-1 → L / H → L+1
S4	389	0.13	H-1 → L+1
S12	334	0.19	H → L+6
**2**			
S1	538	0.57	H → L
S8	443	0.21	H → L+2
S10	427	0.56	H-1 → L+2
S13	415	0.23	H-2 → L+2
S14	400	0.71	H-3 → L+1
**FGNR-tetramer**			
S1	600	1.01	H → L
S6	516	0.33	H-1 → L+1
S8	505	0.09	H-2 → L / H → L+3
S10	488	0.15	H-3 → L
S20	456	1.41	H-3 → L+2

11. Terahertz spectroscopic study of FGNR

Ultrafast optical pump−terahertz probe (OPTP) spectroscopy was employed to measure the photoconductivity of **FGNR**. The working principle of OPTP spectroscopy and its applicability for unveiling the charge transporting properties in nanostructured materials have been widely reported previously.[9-11]

Drude-Smith (DS) model and fitting

The Drude-Smith (DS) model, modified from the classic Drude model, has been employed intensively to characterize the charge transport properties in nanostructured semiconductors (such as graphene nanoribbons[12, 13]). In the DS model, a parameter *c* is introduced to characterize the backscattering

probability, e.g., due to structural confinement. The frequency-resolved conductivity spectra of **FGNR** fitted by the DS model using the expression:

$$\sigma(\omega) = \frac{\omega_p^2 \varepsilon_0 \tau}{1 - \mathrm{i}\omega\tau}\left(1 + \frac{c}{1 - \mathrm{i}\omega\tau}\right)$$

where τ, ω_p and ε_0 are the charge scattering time, the plasma frequency, and vacuum permittivity, respectively.

Reduced mass of photogenerated charge carriers

Due to the presence of *tert*-butyl substituents at the peripheral sites which induces steric hindrance, the geometry of **FGNR** is distorted, with a tilt angle of the edge phenyl groups of about 40-45°, as shown in the unit cell (Figure S18) that applied for the calculation of infinite **FGNR**. This, in turn, affects the transport properties of **FGNR**. From the band dispersion, which are reported in Figure S19, we computed the reduced mass around the VB maximum (VBM) and CB minimum (CBM), as well as the VBM and CBM orbitals. As result, we obtained a value of 0.95 for both m_h* (effective mass of the electron) and m_e* (effective mass of the hole). Furthermore, we estimated the effective reduced mass of charge carriers m*, by considering the averaged values for both charges, with the equation: $\frac{1}{m^*} = \frac{1}{m_e^*} + \frac{1}{m_h^*}$. The intrinsic charge mobility μ $(= e\tau\ /\ m^*)$ was estimated to be 104 ± 3 $cm^2\ V^{-1}\ s^{-1}$. Note that, this mobility value is slightly lower than GNRs with more planar structure and similar charge scattering time (20~30 fs).[13, 14] This is mainly due to a relatively large effective mass of the charge carriers in **FGNR**, originating from its non-planar geometry.

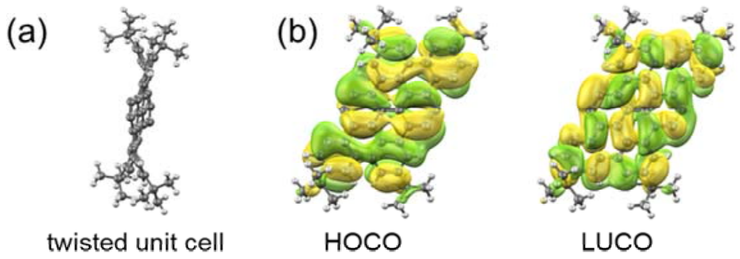

Figure S18. (a)Unit cell for the calculation of infinite **FGNR**. (b) Highest occupied crystalline orbitals (HOCO) and lowest unoccupied crystalline orbitals (LUCO) of the unit cell of **FGNR**.

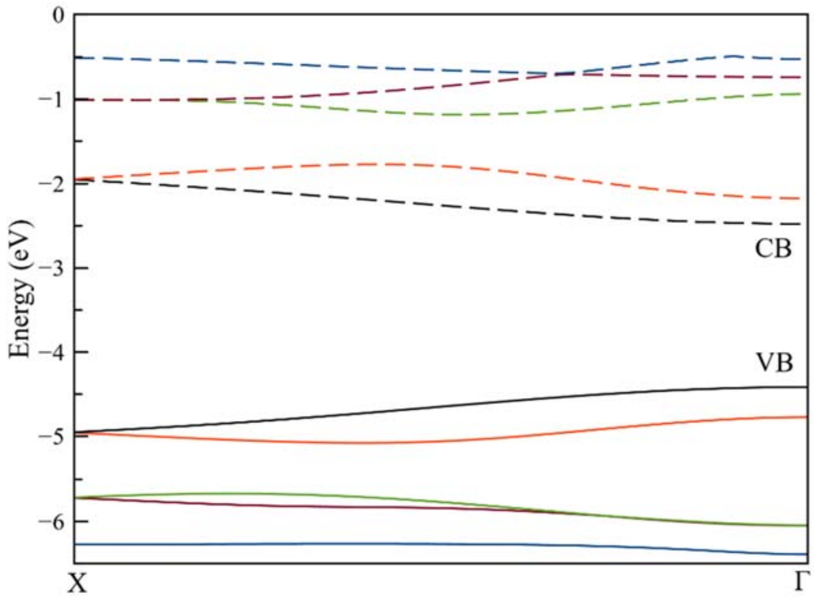

Figure S19. Calculated band structure of **FGNR**.

12. NMR spectra

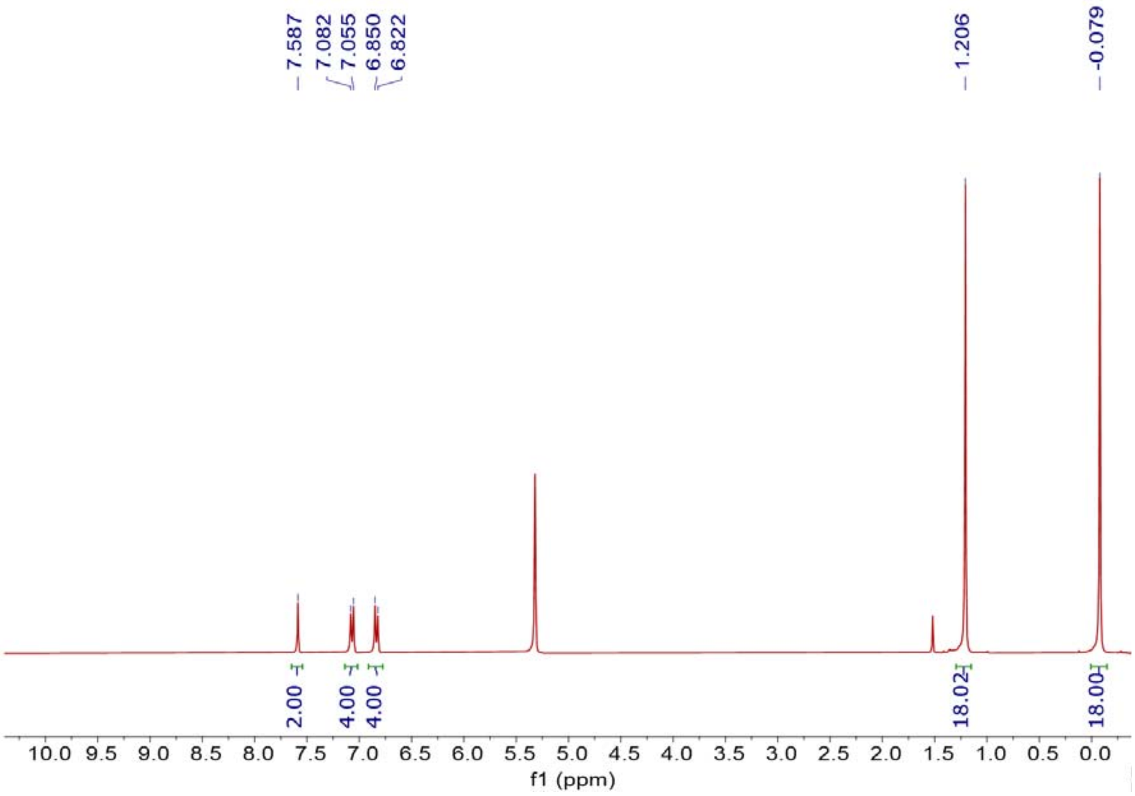

Figure S20. ^{1}H NMR spectrum of compound **12** (300 MHz, CD_2Cl_2, 298 K).

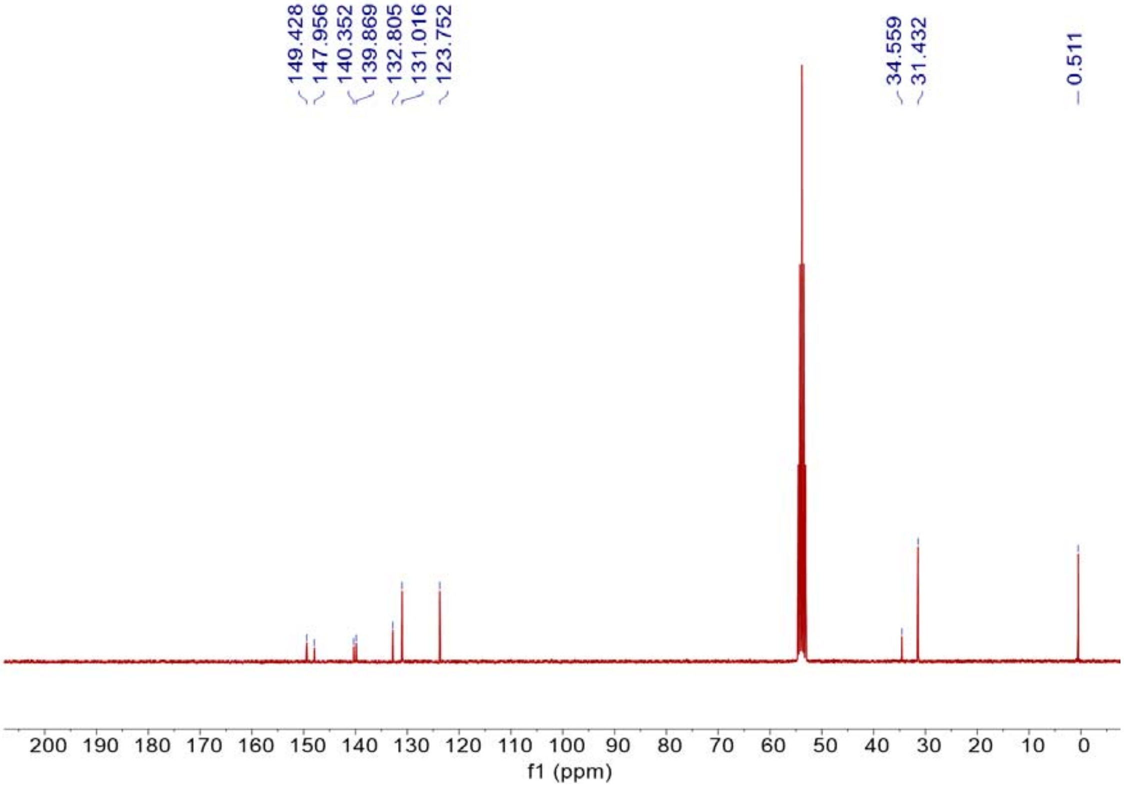

Figure S21. ^{13}C NMR spectrum of compound **12** (75 MHz, CD_2Cl_2, 298 K).

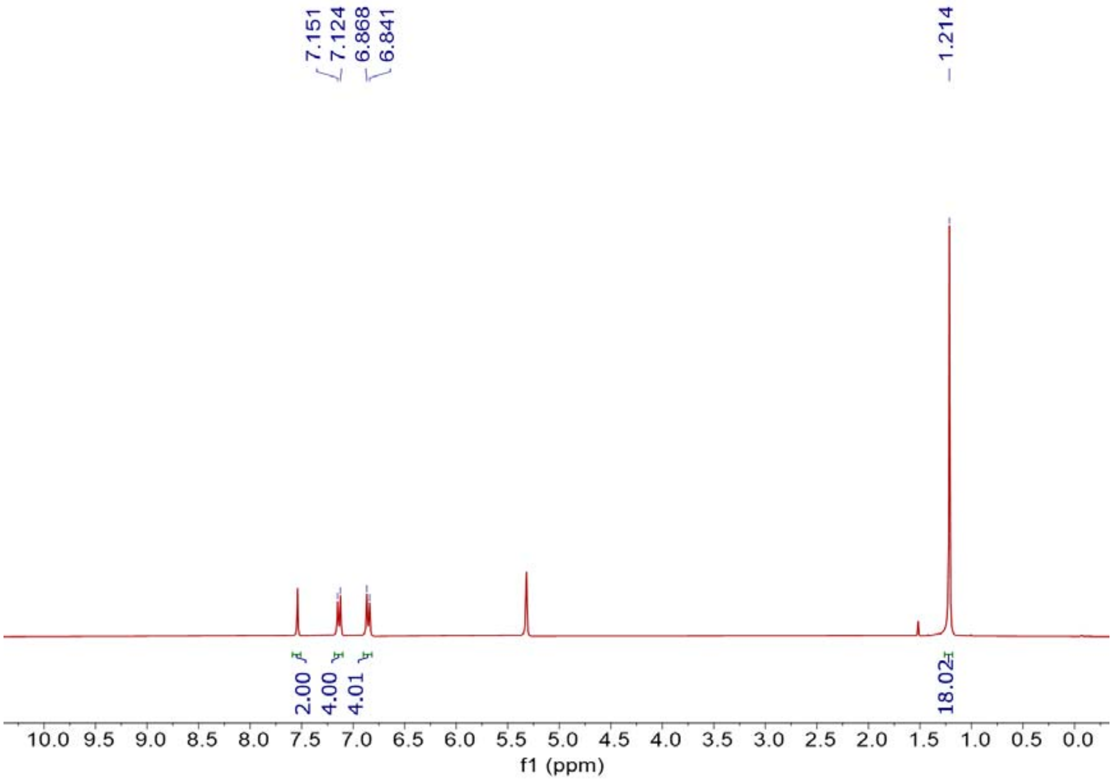

Figure S22. ^{1}H NMR spectrum of compound **3** (300 MHz, CD_2Cl_2, 298 K).

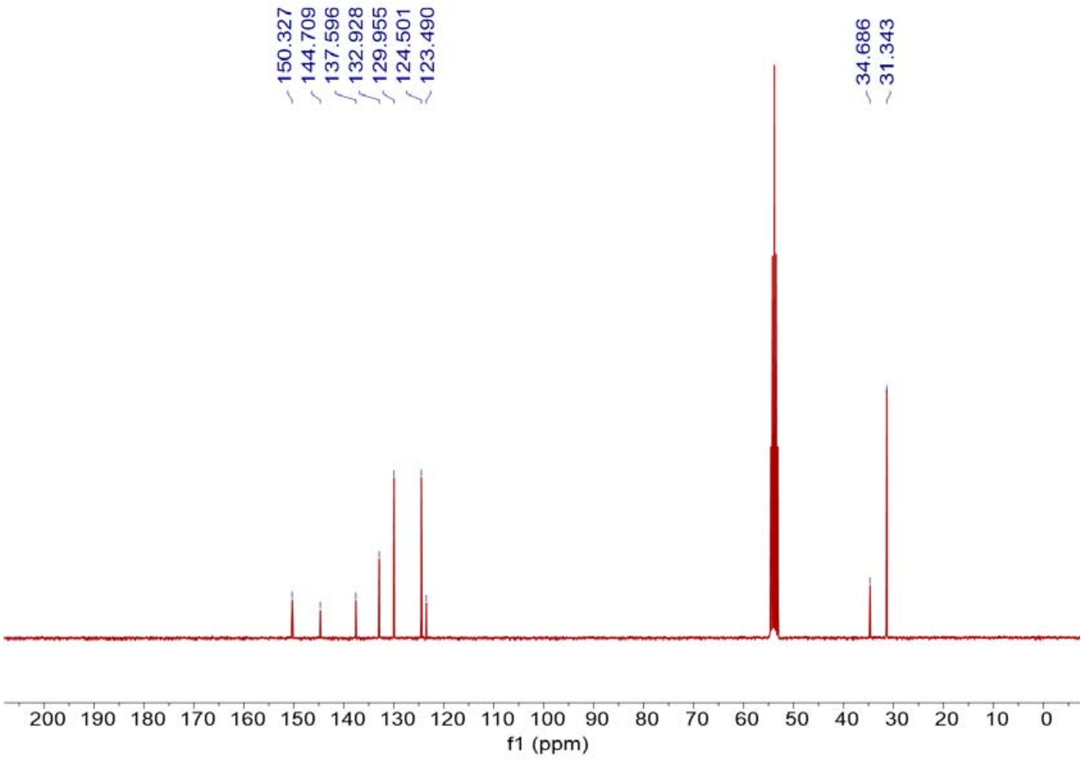

Figure S23. ^{13}C NMR spectrum of compound **3** (75 MHz, CD_2Cl_2, 298 K).

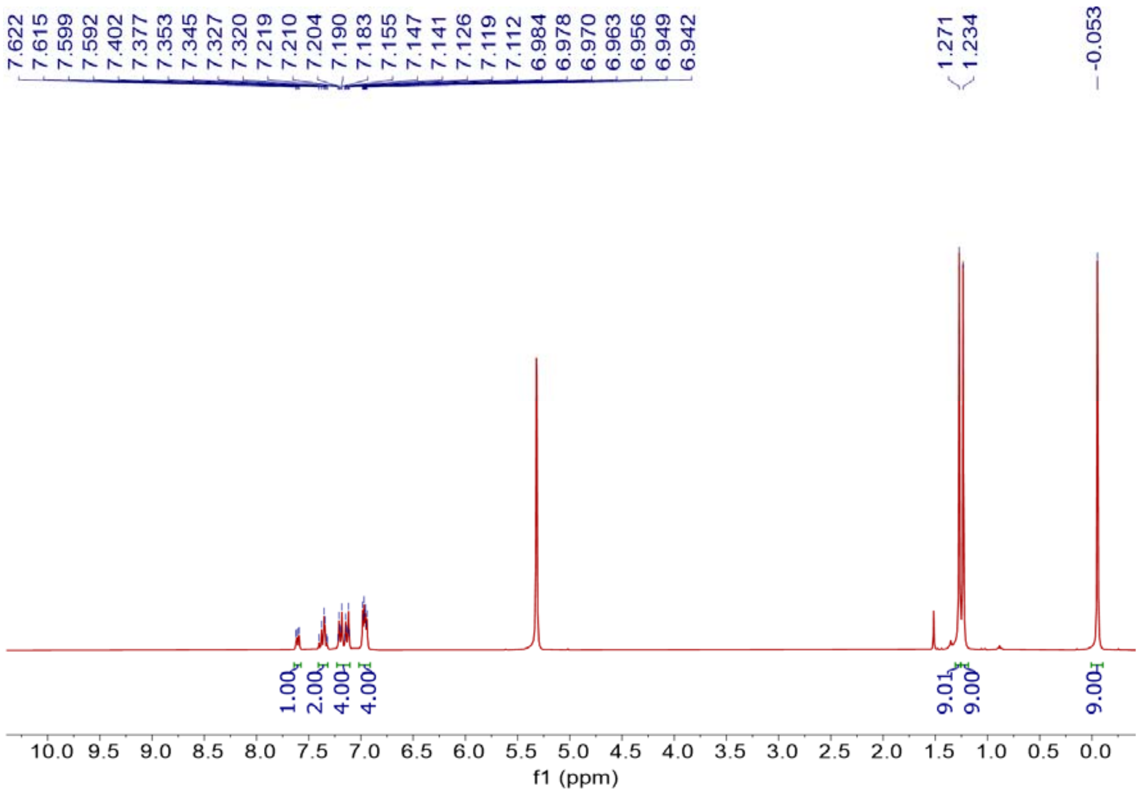

Figure S24. ^{1}H NMR spectrum of compound **14** (300 MHz, CD_2Cl_2, 298 K).

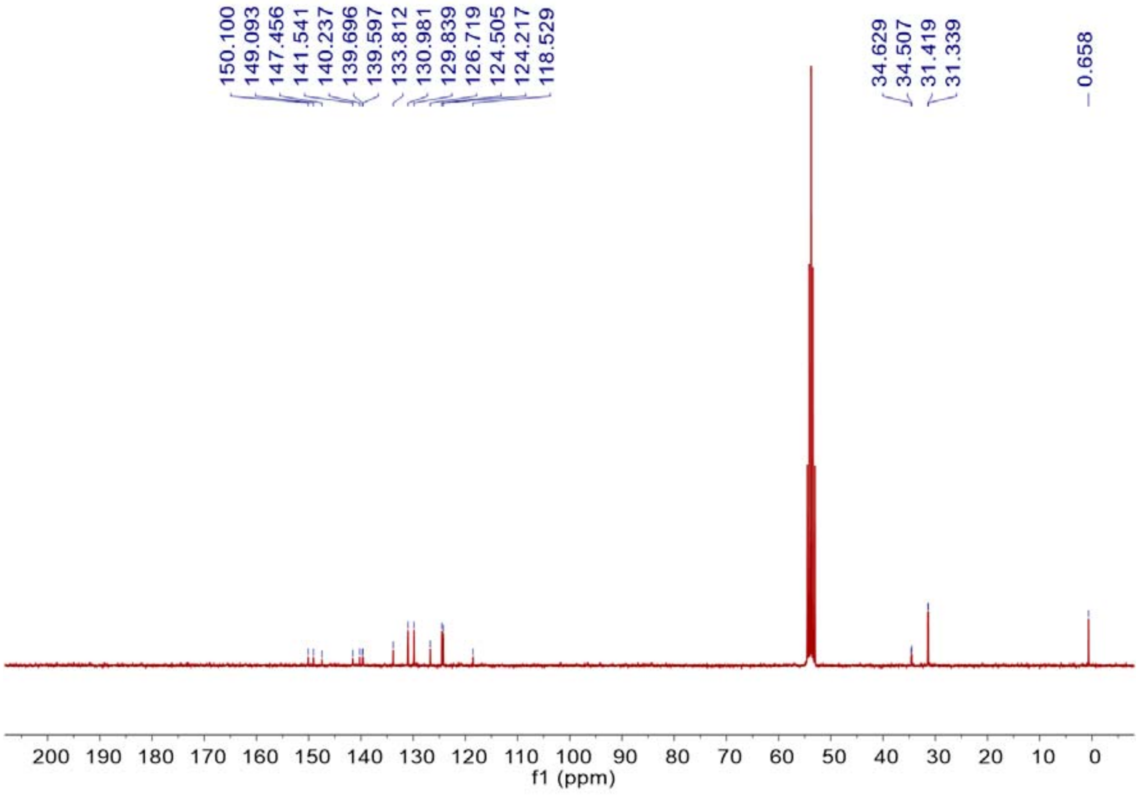

Figure S25. ^{13}C NMR spectrum of compound **14** (75 MHz, CD_2Cl_2, 298 K).

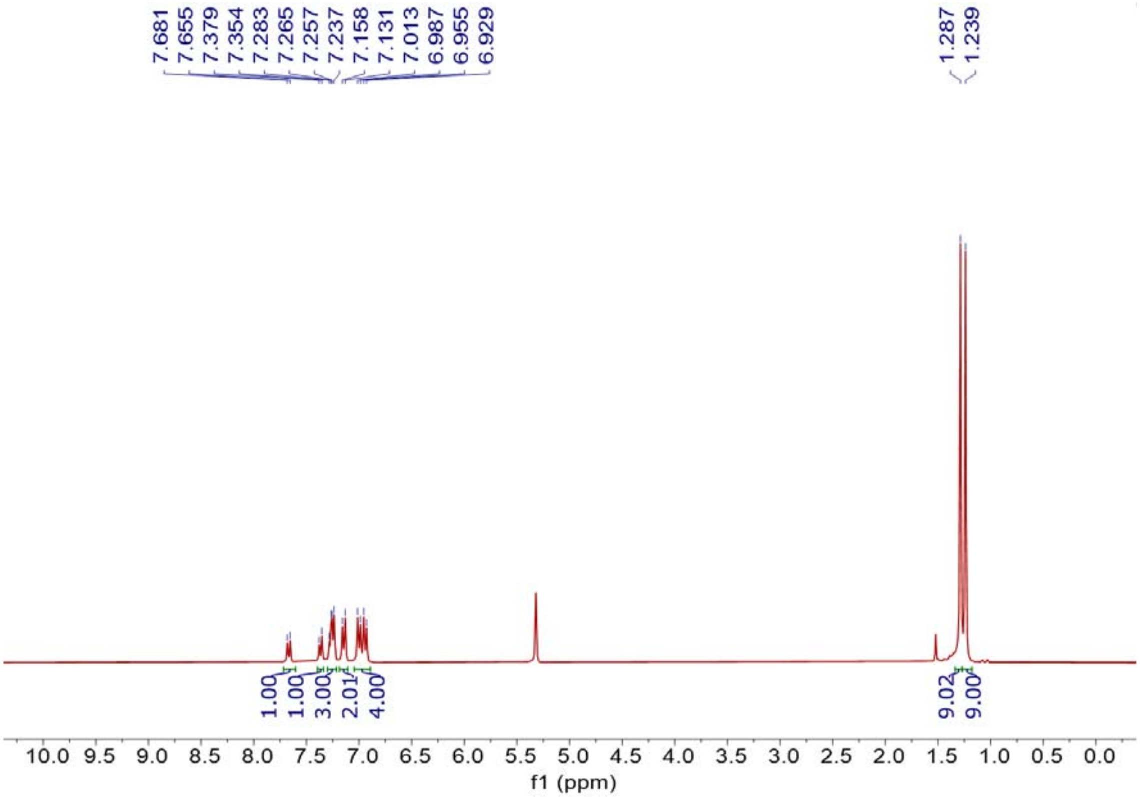

Figure S26. ^{1}H NMR spectrum of compound **15** (300 MHz, CD_2Cl_2, 298 K).

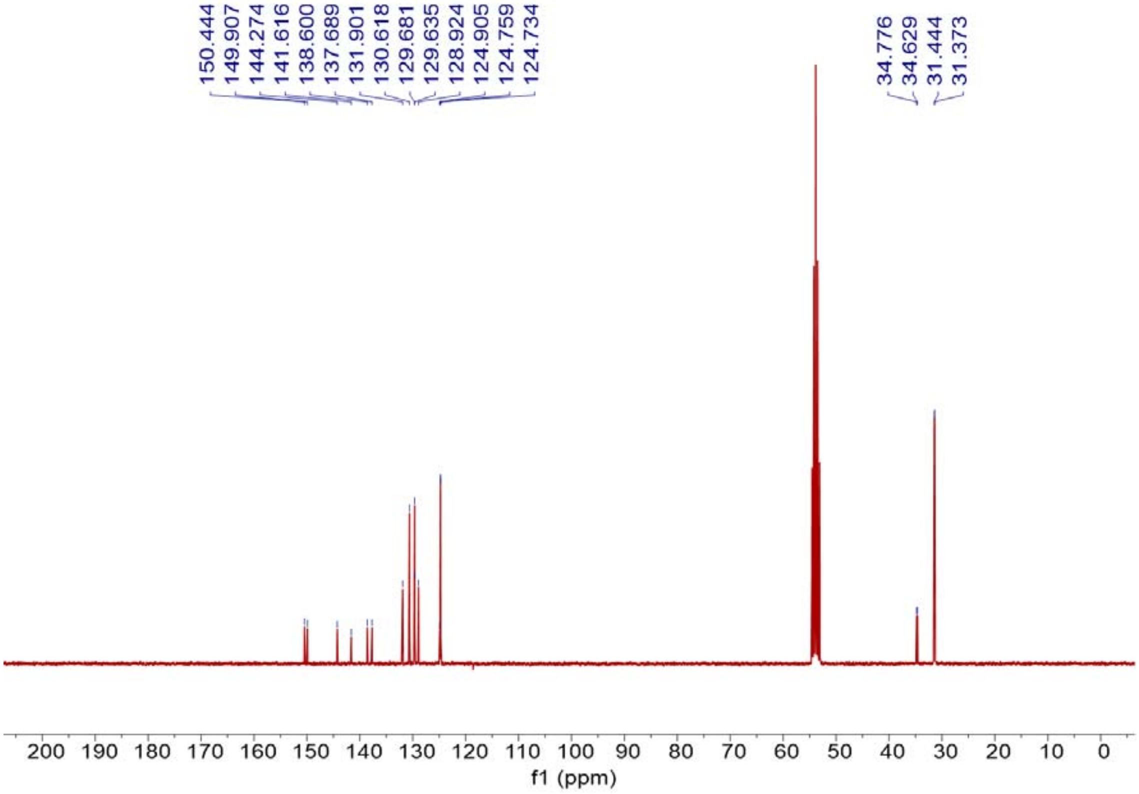

Figure S27. ^{13}C NMR spectrum of compound **15** (75 MHz, CD_2Cl_2, 298 K).

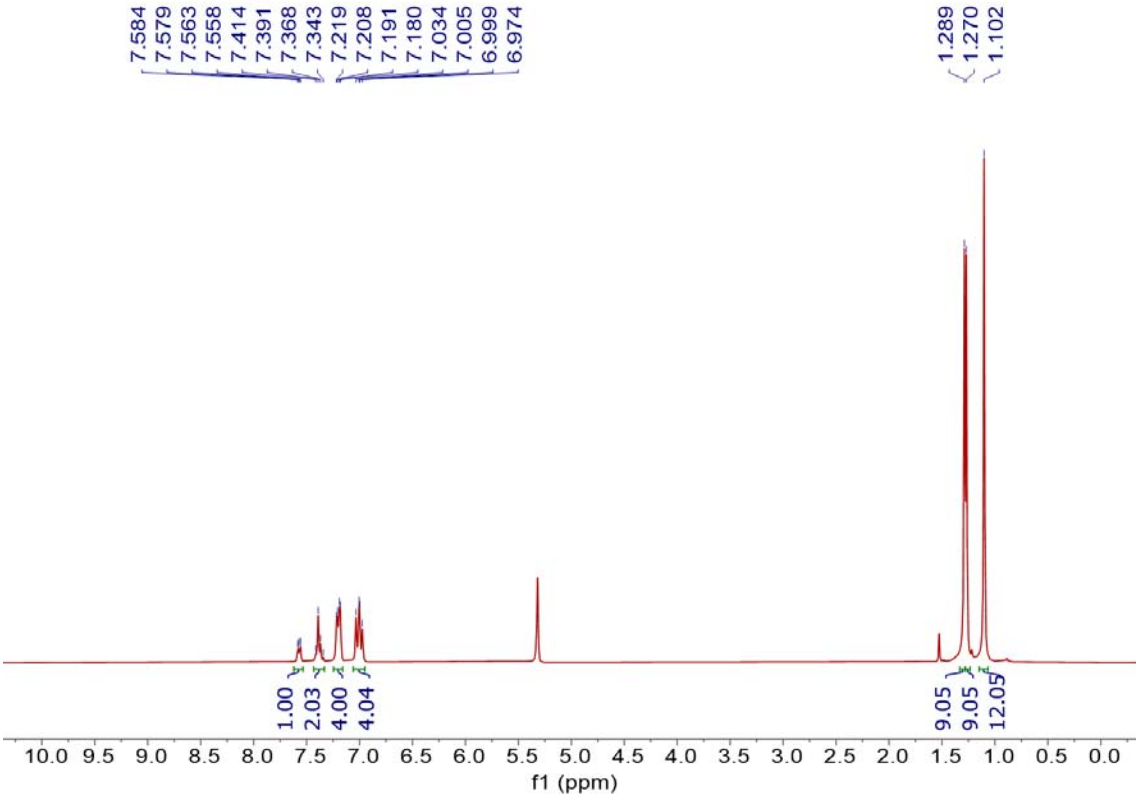

Figure S28. ^{1}H NMR spectrum of compound **4** (300 MHz, CD_2Cl_2, 298 K).

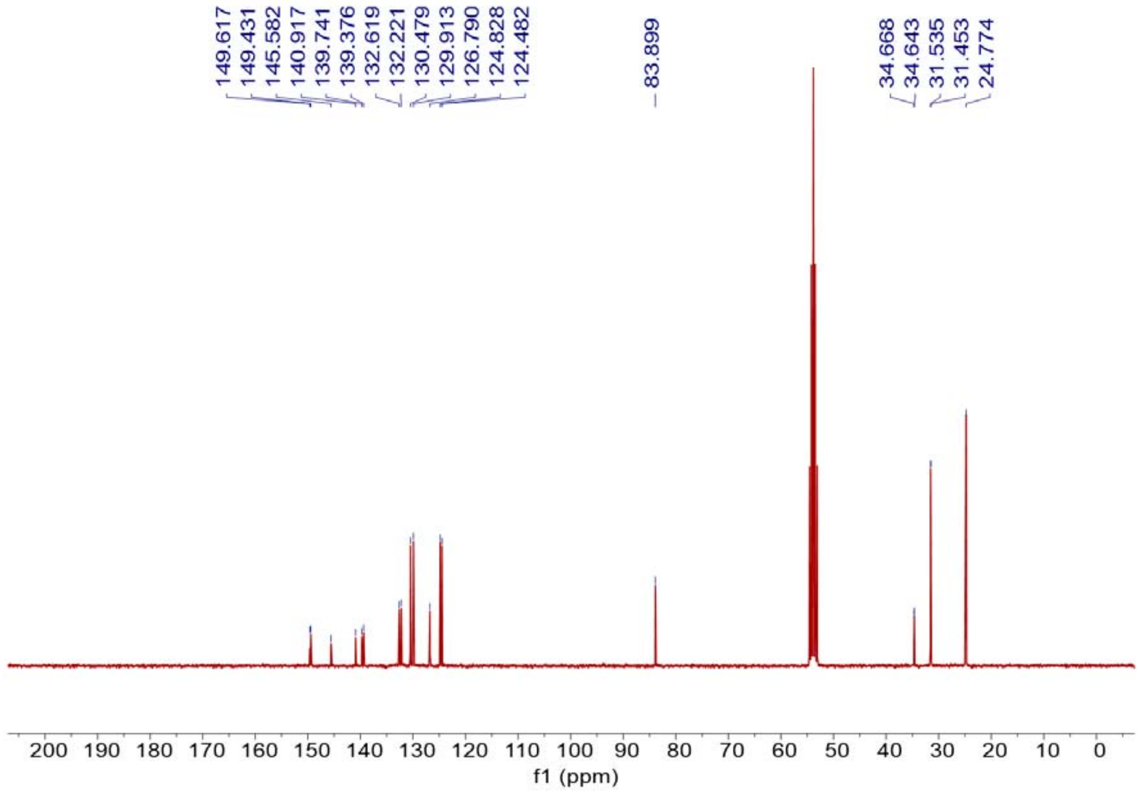

Figure S29. ^{13}C NMR spectrum of compound **4** (75 MHz, CD_2Cl_2, 298 K).

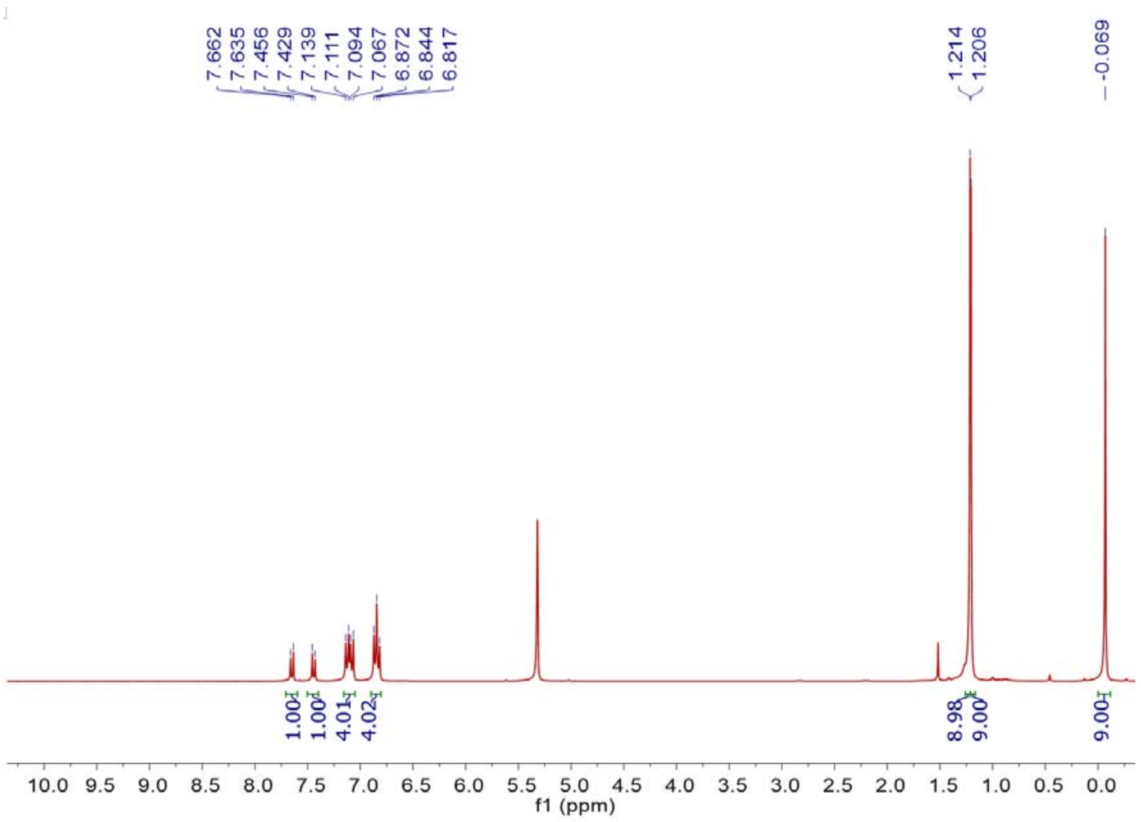

Figure S30. ^{1}H NMR spectrum of compound **16** (300 MHz, CD_2Cl_2, 298 K).

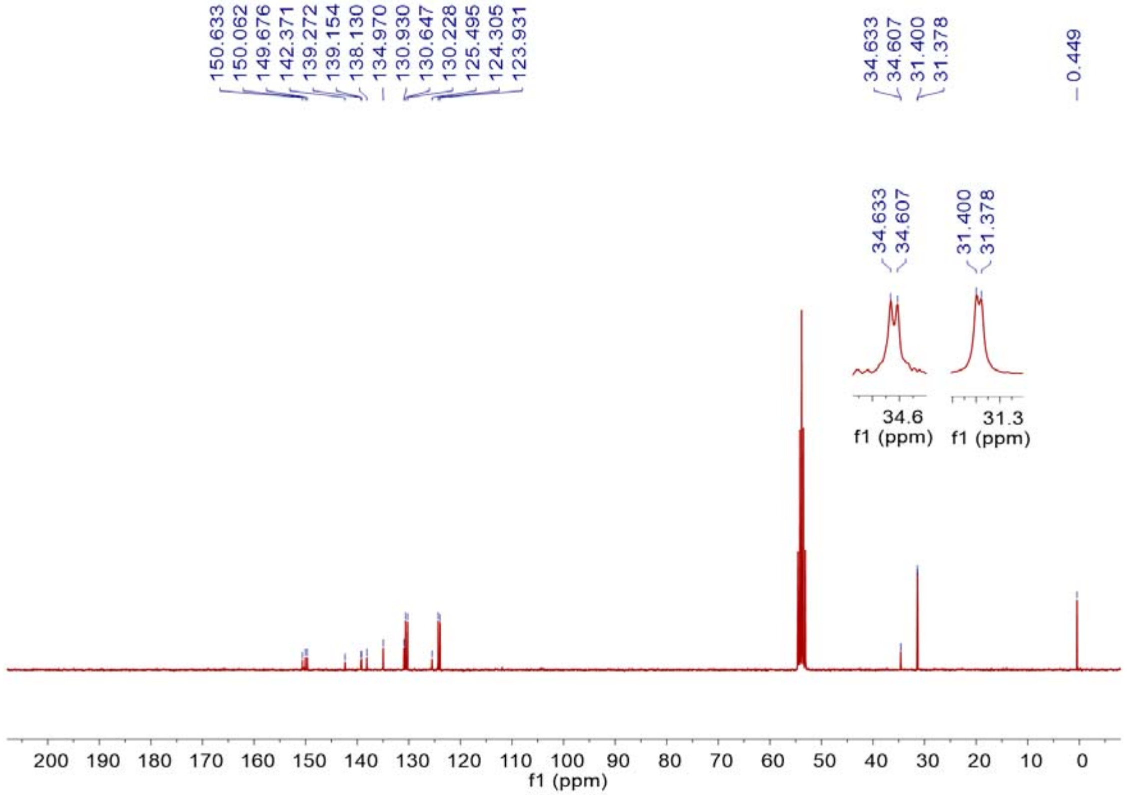

Figure S31. ^{13}C NMR spectrum of compound **16** (75 MHz, CD_2Cl_2, 298 K).

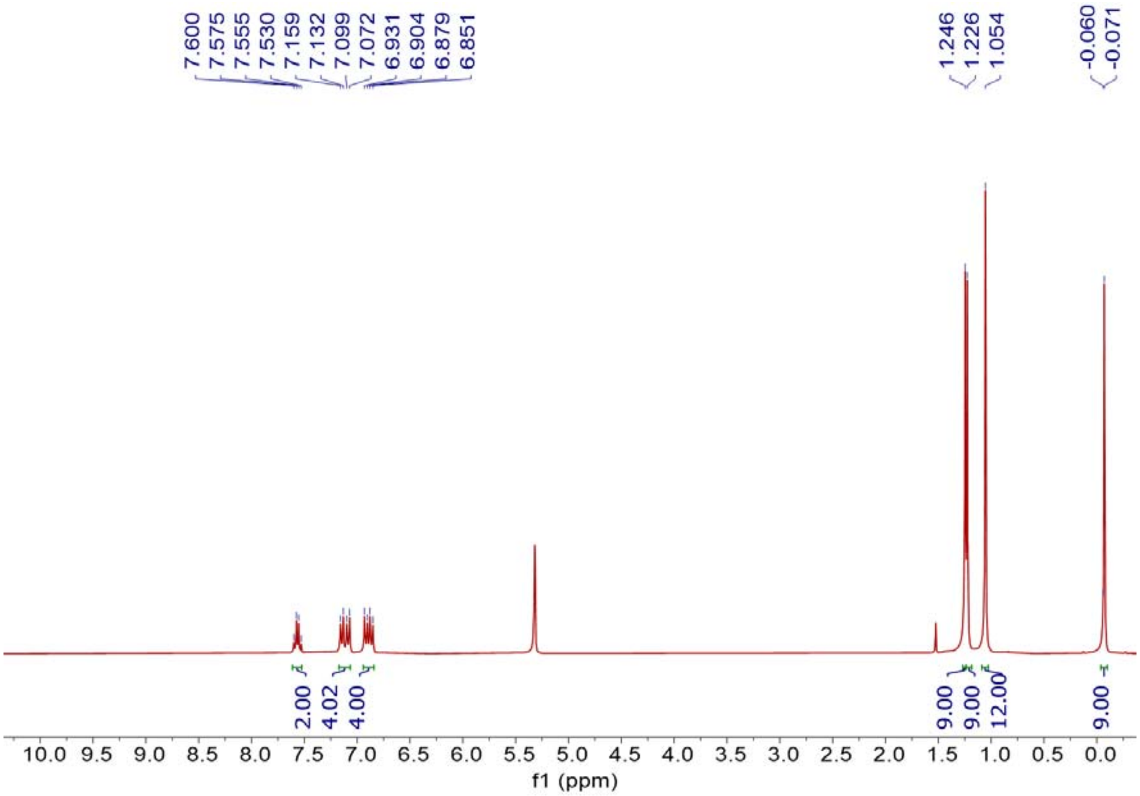

Figure S32. ^{1}H NMR spectrum of compound **6** (300 MHz, CD_2Cl_2, 298 K).

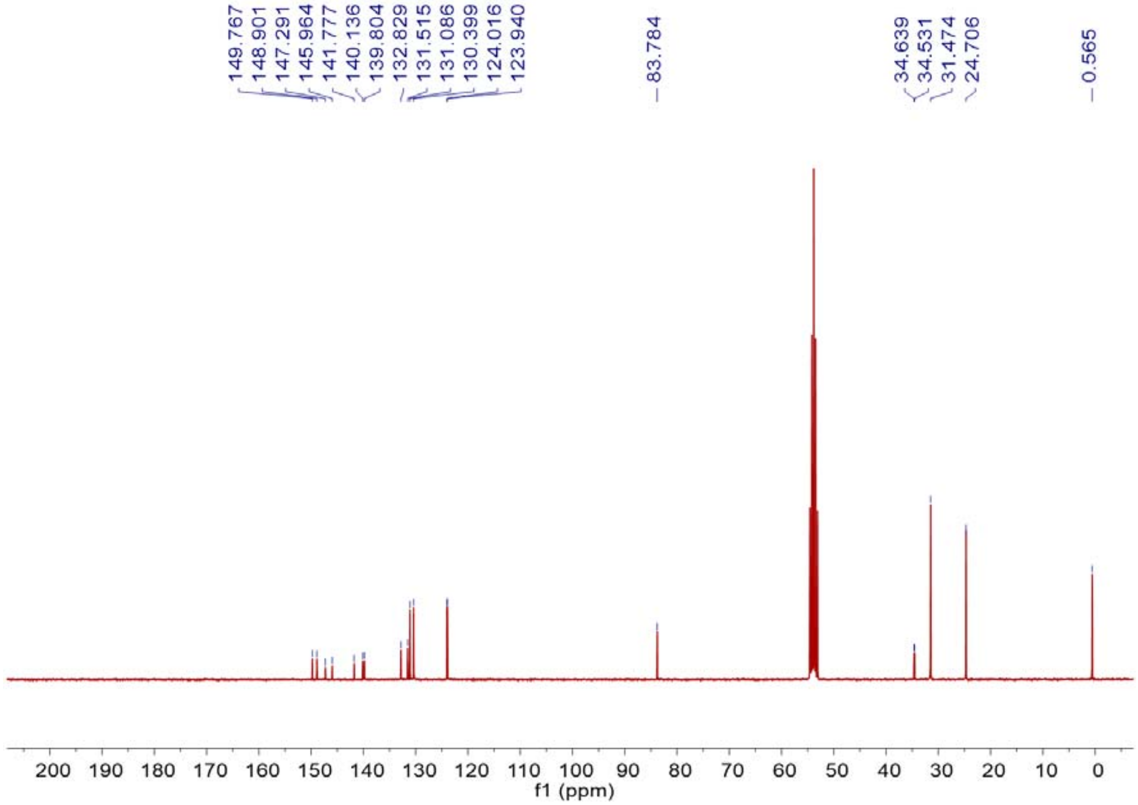

Figure S33. ^{13}C NMR spectrum of compound **6** (75 MHz, CD_2Cl_2, 298 K).

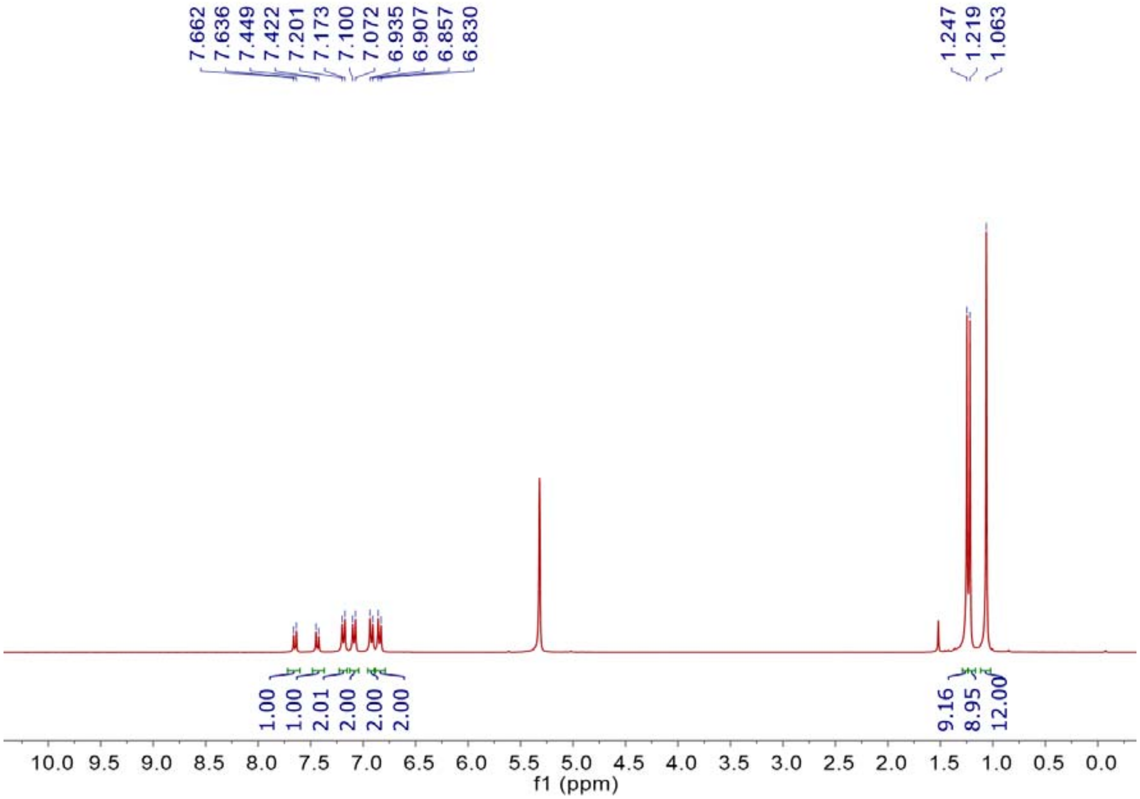

Figure S34. ^{1}H NMR spectrum of compound **10** (300 MHz, CD_2Cl_2, 298 K).

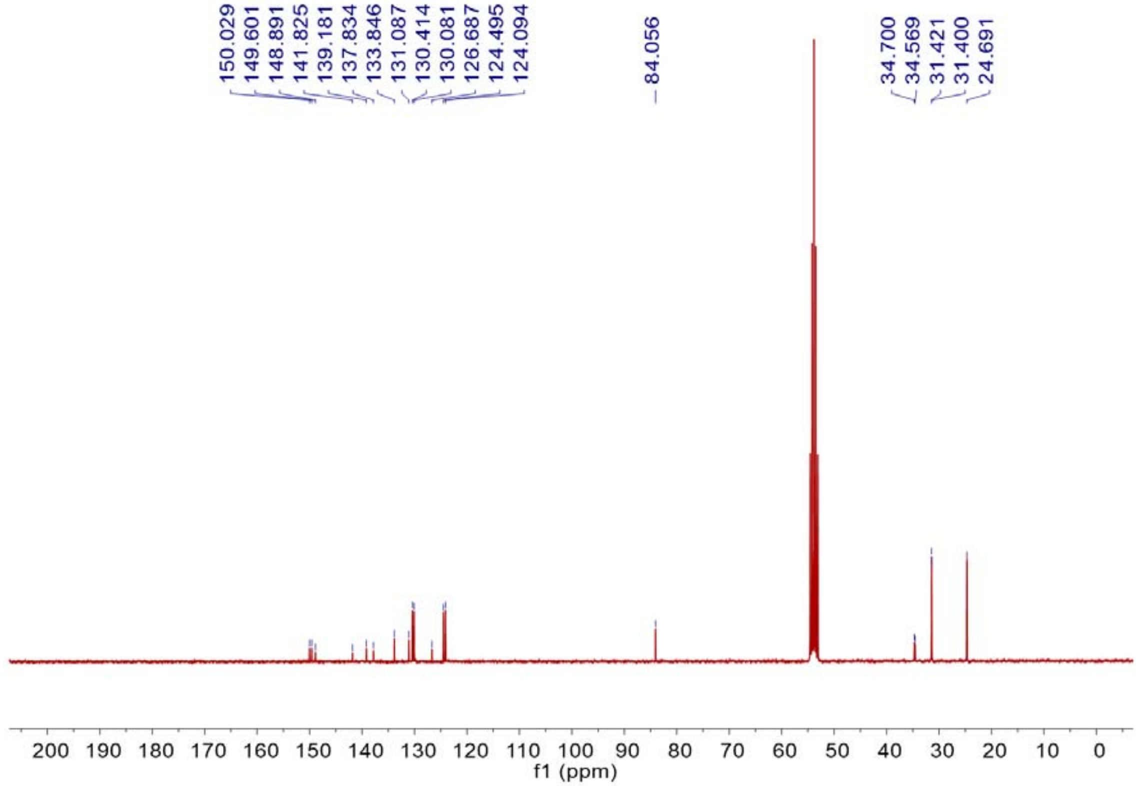

Figure S35. ^{13}C NMR spectrum of compound **10** (75 MHz, CD_2Cl_2, 298 K).

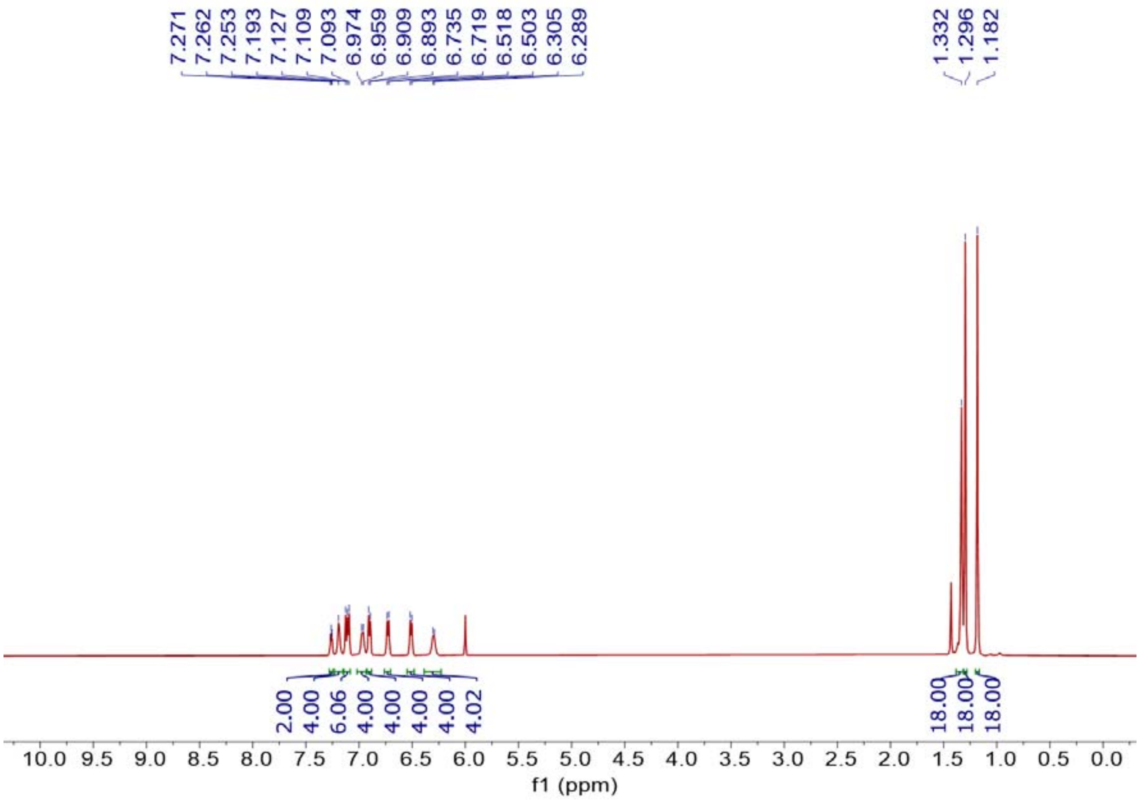

Figure S36. ^{1}H NMR spectrum of compound **5** (500 MHz, $C_2D_2Cl_4$, 393 K).

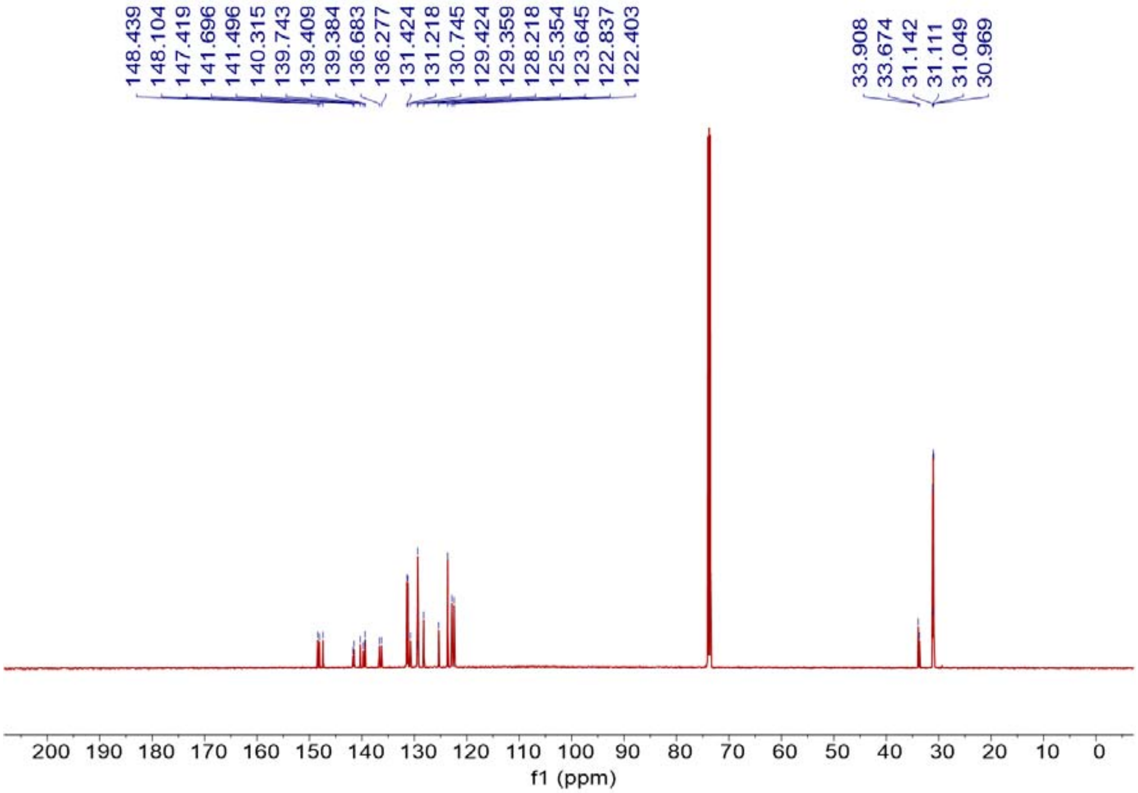

Figure S37. ^{13}C NMR spectrum of compound **5** (125 MHz, $C_2D_2Cl_4$, 393 K).

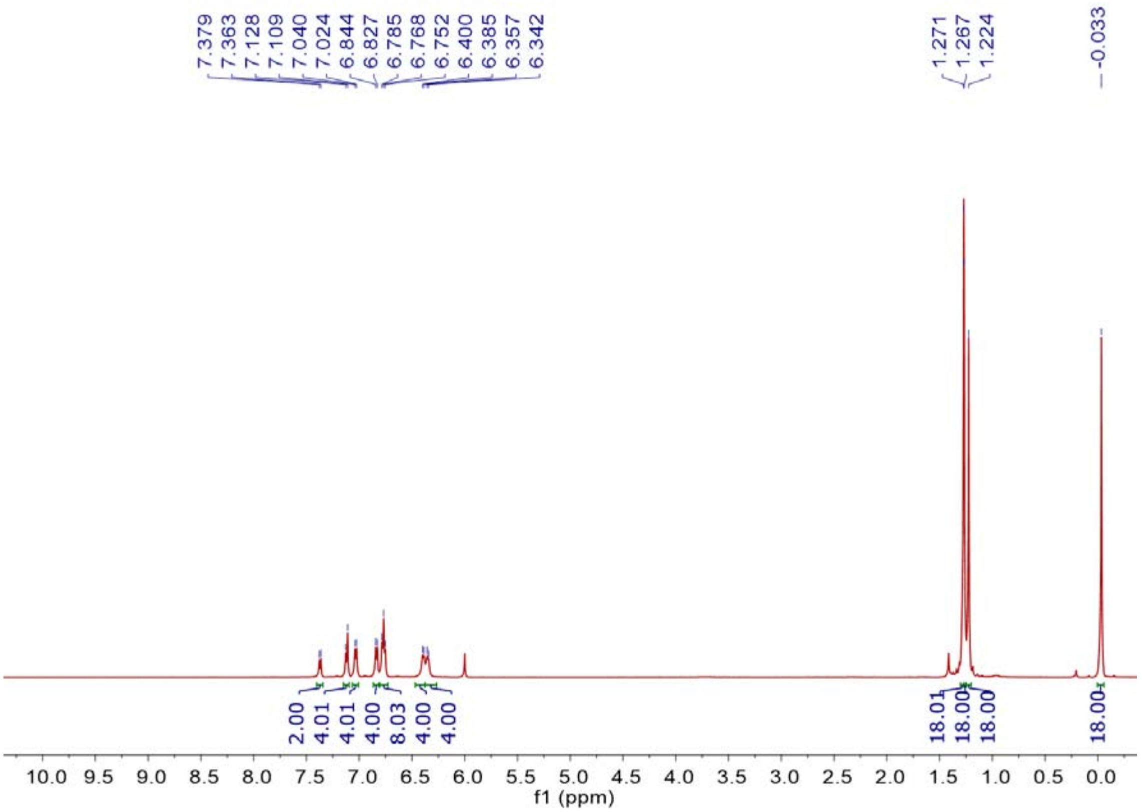

Figure S38. ^{1}H NMR spectrum of compound **7** (500 MHz, $C_2D_2Cl_4$, 393 K).

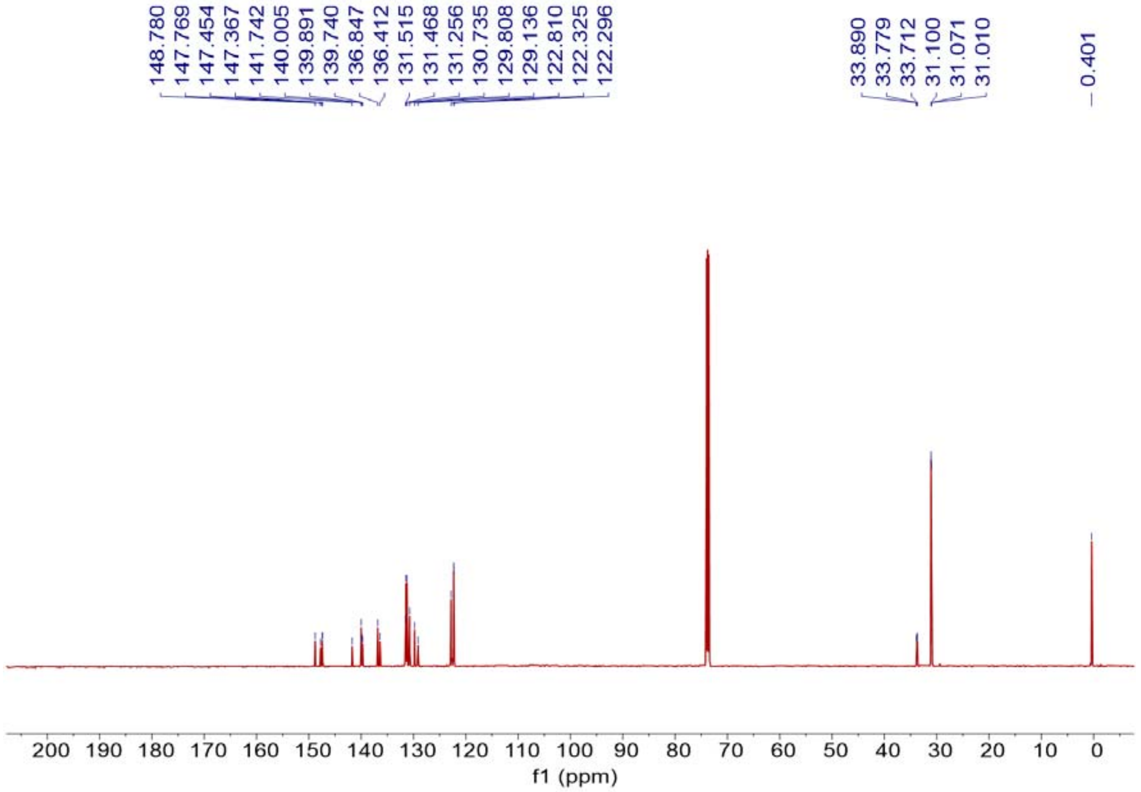

Figure S39. ^{13}C NMR spectrum of compound **7** (125 MHz, $C_2D_2Cl_4$, 393 K).

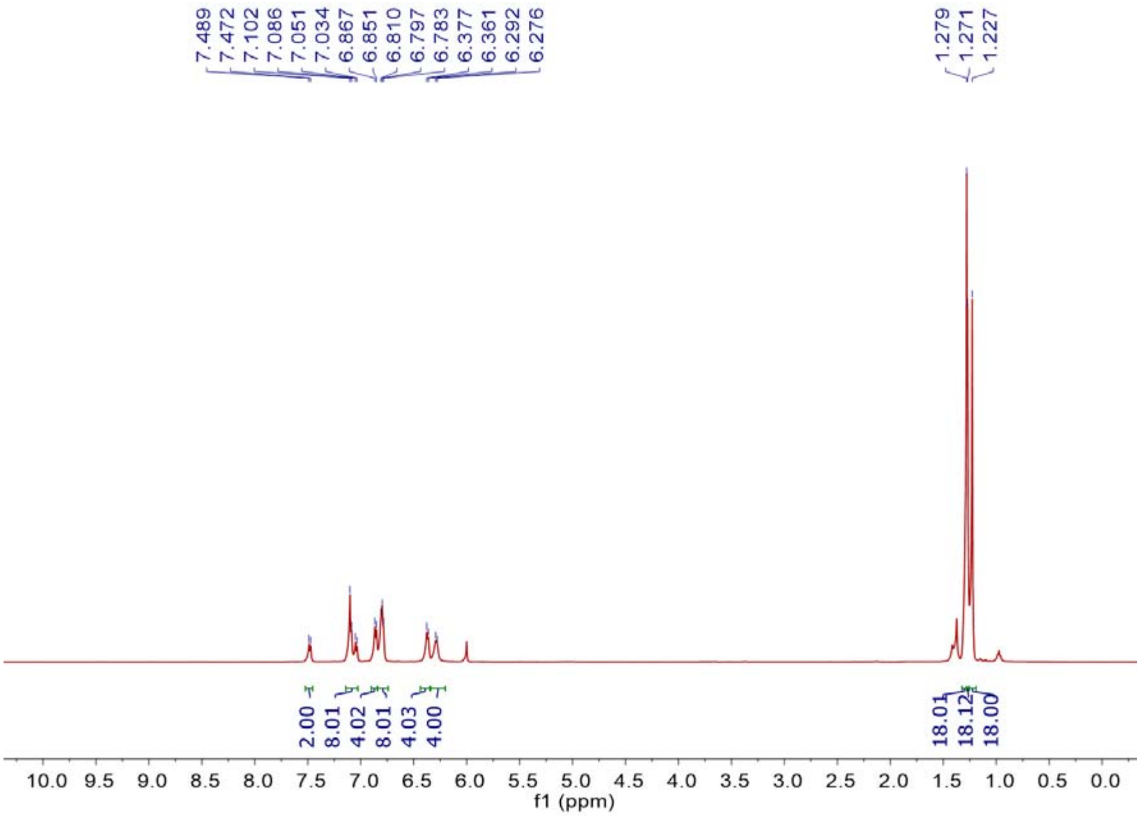

Figure S40. ^{1}H NMR spectrum of compound **8** (500 MHz, $C_2D_2Cl_4$, 393 K).

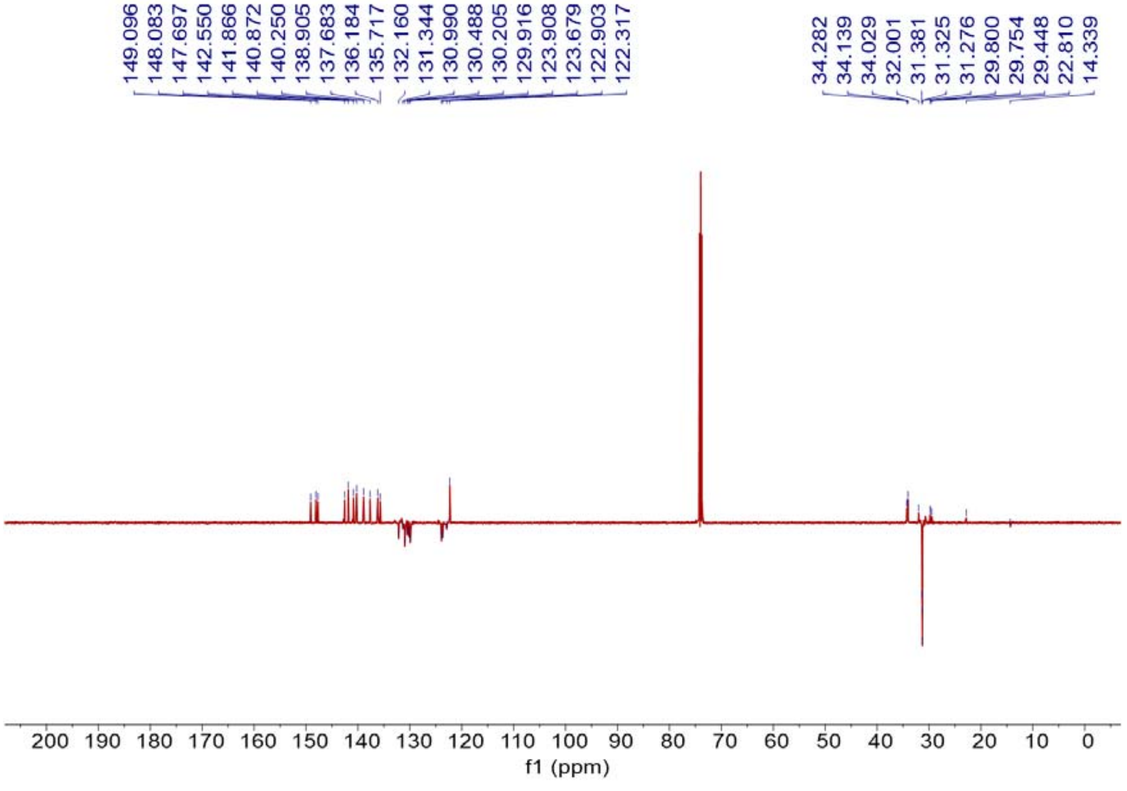

Figure S41. ^{13}C NMR spectrum of compound **8** (125 MHz, $C_2D_2Cl_4$, 393 K).

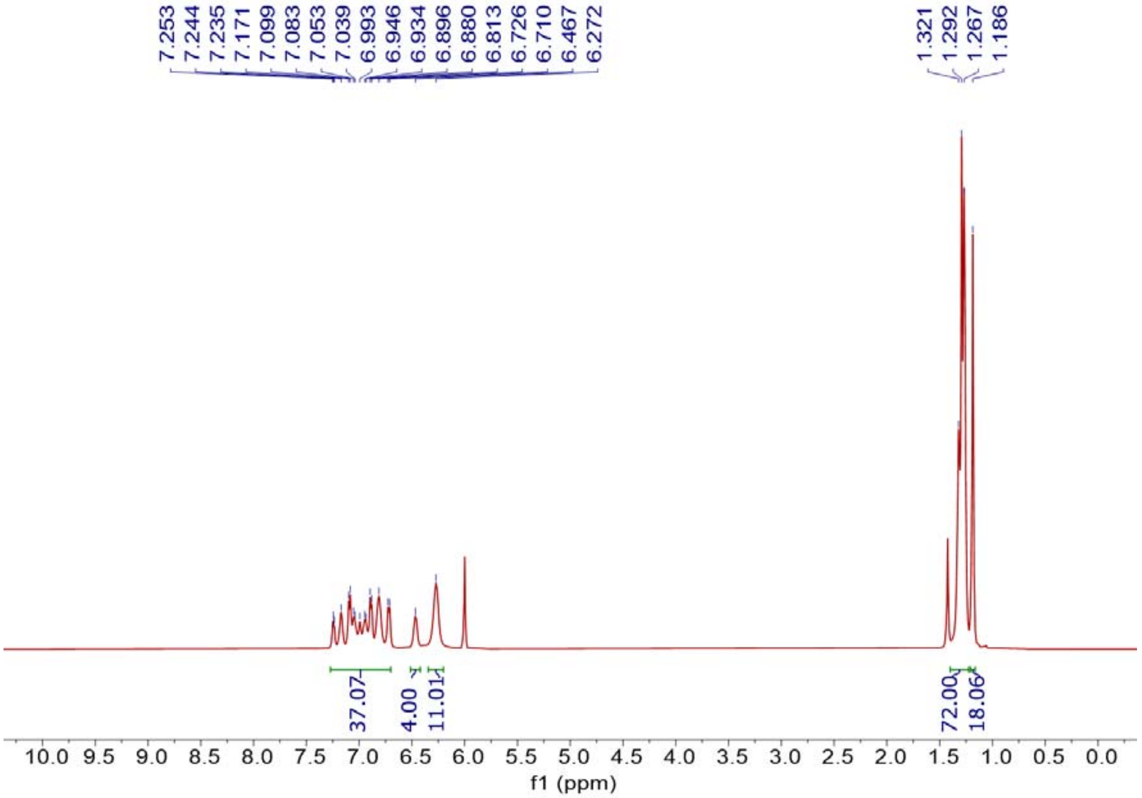

Figure S42. ^{1}H NMR spectrum of compound **9** (500 MHz, $C_2D_2Cl_4$, 393 K).

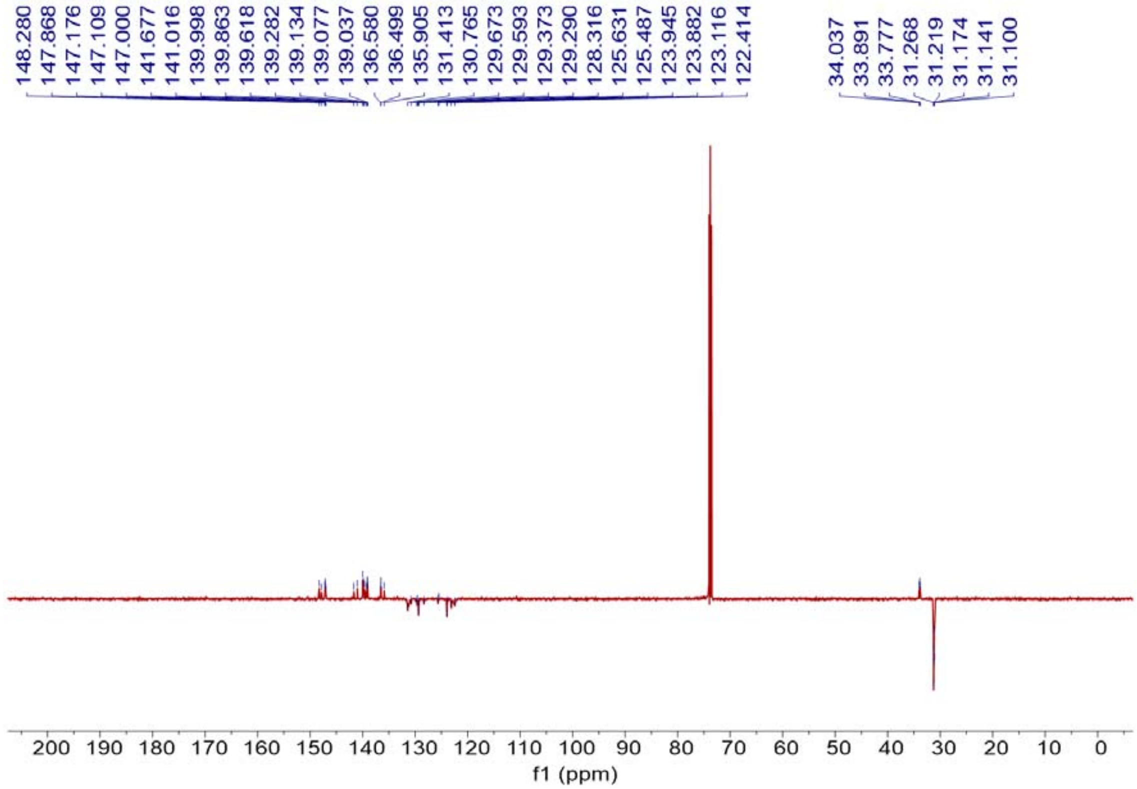

Figure S43. ^{13}C NMR spectrum of compound **9** (125 MHz, $C_2D_2Cl_4$, 393 K).

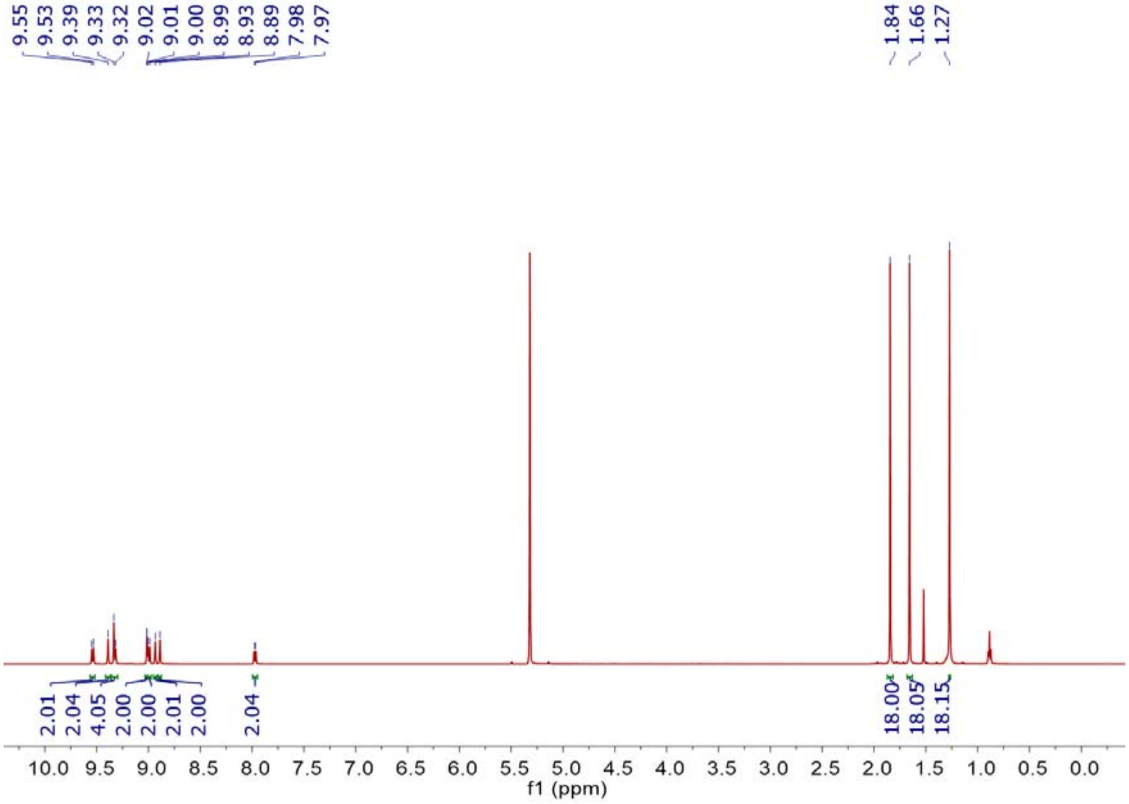

Figure S44. ^{1}H NMR of **1**. (500 MHz, CD_2Cl_2, 298 K).

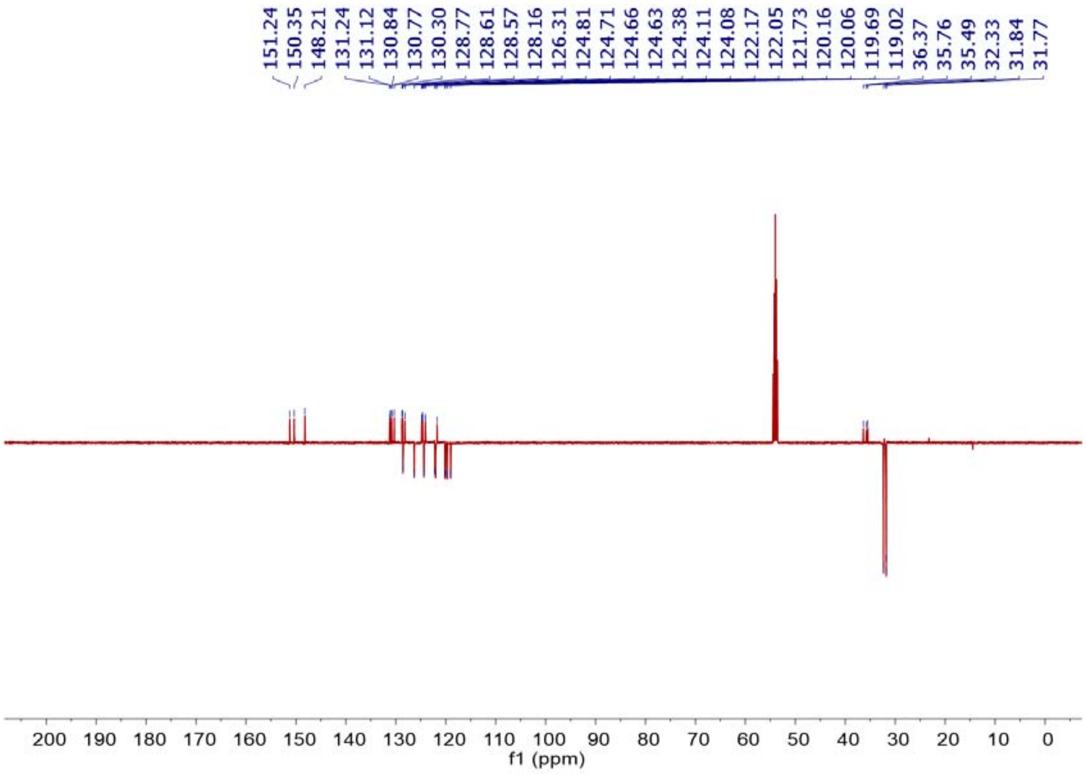

Figure S45. ^{13}C NMR of **1** (125 MHz, CD_2Cl_2, 298 K).

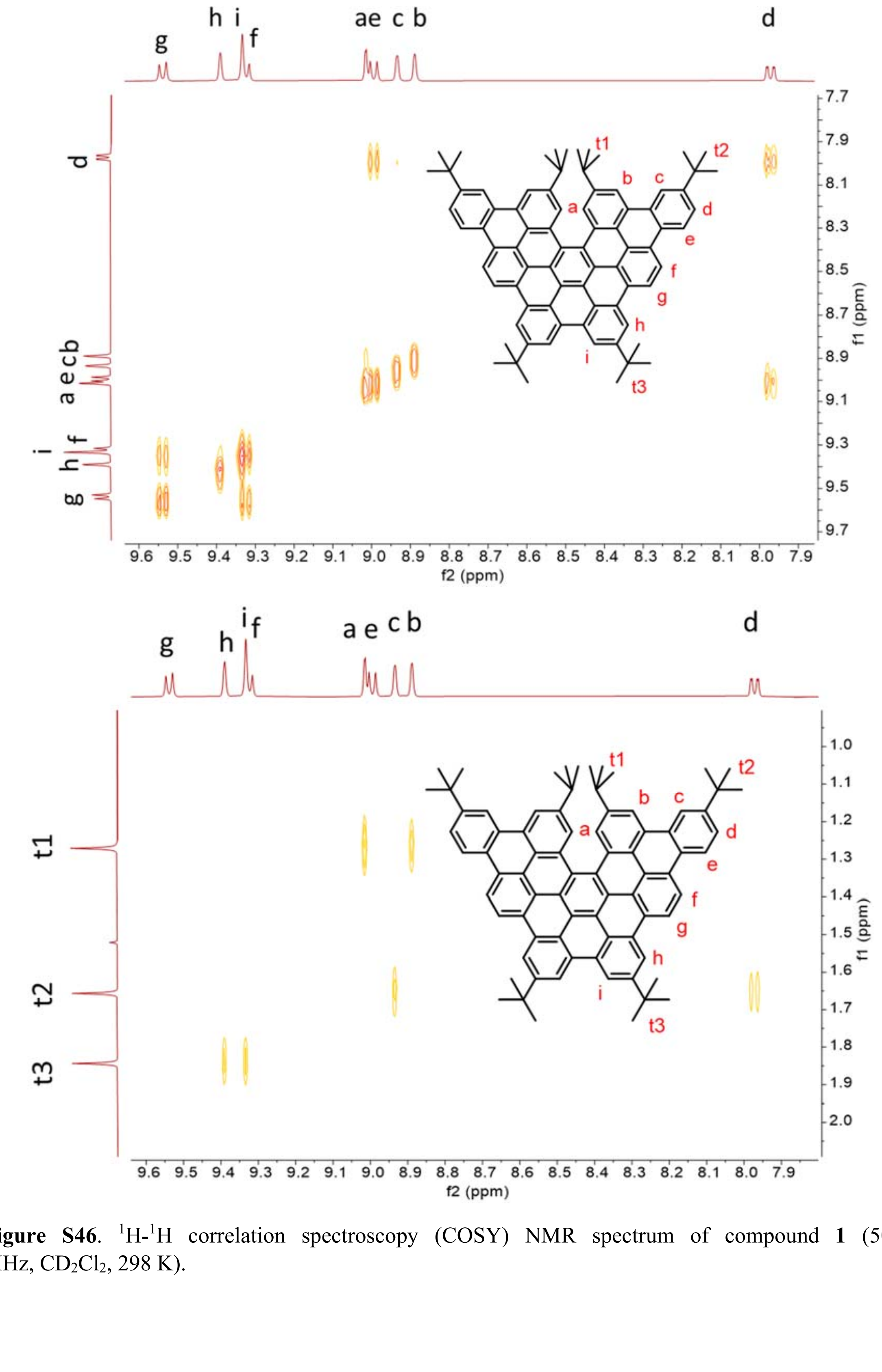

Figure S46. ^{1}H-^{1}H correlation spectroscopy (COSY) NMR spectrum of compound **1** (500 MHz, CD_2Cl_2, 298 K).

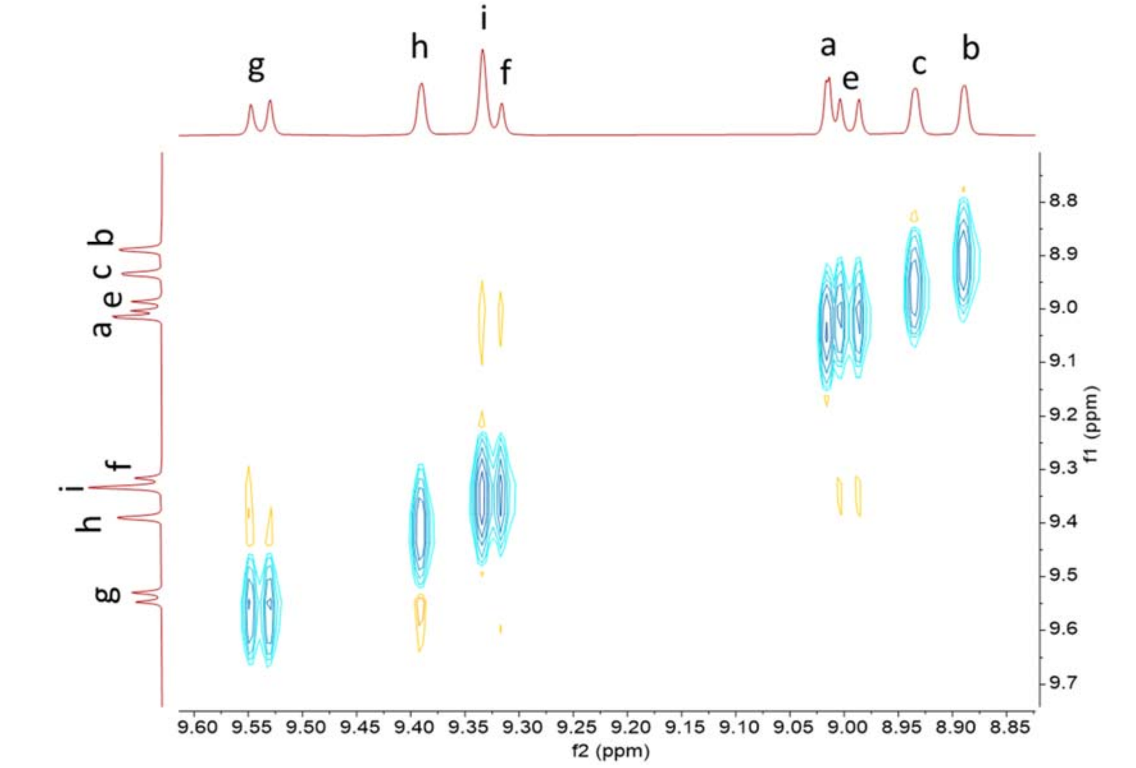

Figure S47. ^{1}H-^{1}H nuclear Overhauser effect spectroscopy (NOESY) NMR spectrum of compound **1** (500 MHz, CD_2Cl_2, 298 K).

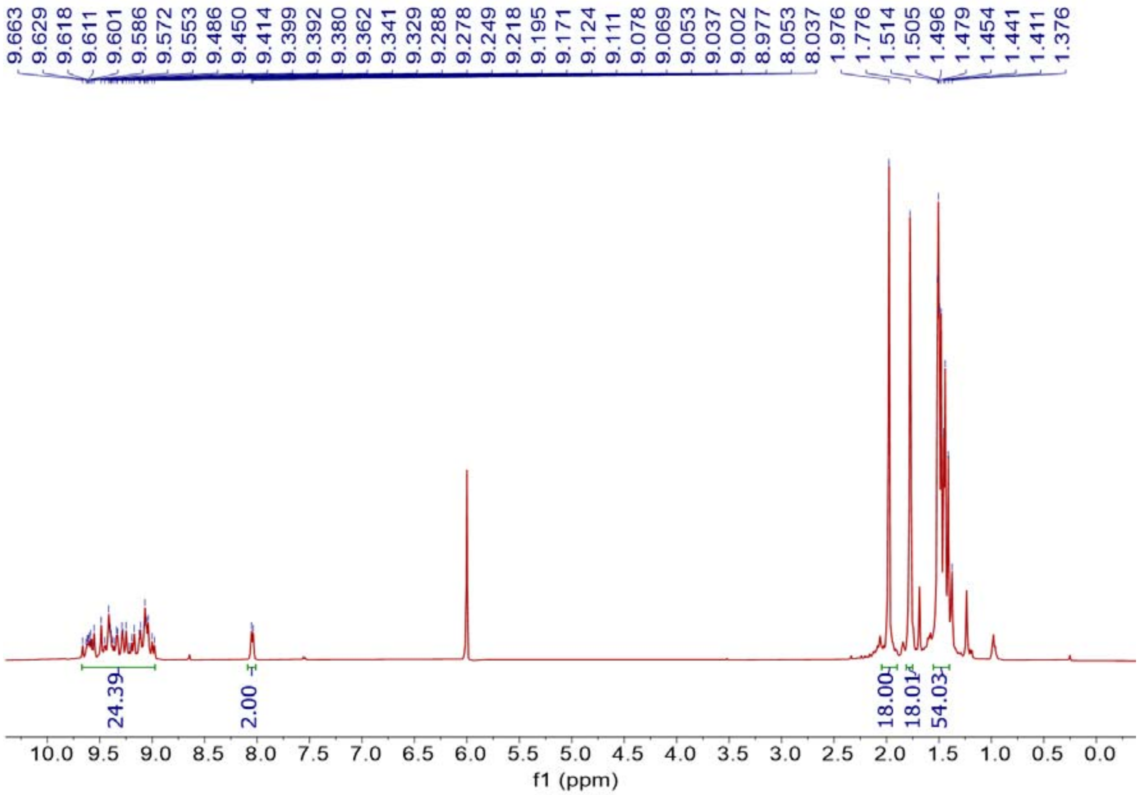

Figure S48. ^{1}H NMR of **2** (500 MHz, $C_2D_2Cl_4$, 393 K).

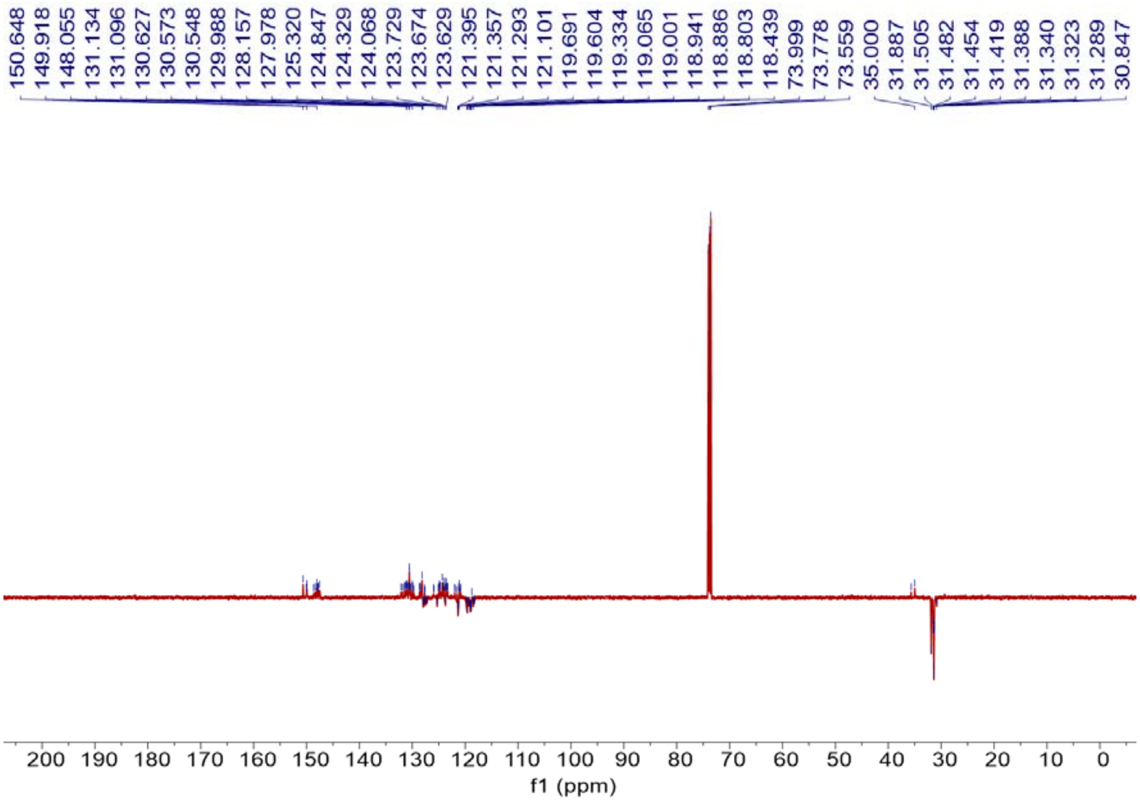

Figure S49. ^{13}C NMR of **2** (125 MHz, $C_2D_2Cl_4$, 393 K).

13. References

(1) Luliński, S.; Serwatowski, J., Regiospecific Metalation of Oligobromobenzenes. *J. Org. Chem.* **2003,** *68*, 5384-5387.

(2) Goedicke, C.; Stegemeyer, H., Resolution and Racemization of Pentahelicene. *Tetrahedron Lett.* **1970,** *11*, 937-940.

(3) Przybilla, L.; Brand, J.-D.; Yoshimura, K.; Räder, H. J.; Müllen, K., MALDI-TOF Mass Spectrometry of Insoluble Giant Polycyclic Aromatic Hydrocarbons by a New Method of Sample Preparation. *Anal. Chem.* **2000,** *72*, 4591-4597.

(4) Cristadoro, A.; Räder, H. J.; Müllen, K., Quantitative Analyses of Fullerene and Polycyclic Aromatic Hydrocarbon Mixtures via Solvent-Free Matrix-Assisted Laser Desorption/Ionization Mass Spectrometry. *Rapid Commun. Mass Spectrom.* **2008,** *22*, 2463-2470.

(5) Yoshimura, K.; Przybilla, L.; Ito, S.; Brand, J. D.; Wehmeir, M.; Räder, H. J.; Müllen, K., Characterization of Large Synthetic Polycyclic Aromatic Hydrocarbons by MALDI- and LD-TOF Mass Spectrometry. *Macromol. Chem. Phys.* **2001,** *202*, 215-222.

(6) Yang, X.; Dou, X.; Rouhanipour, A.; Zhi, L.; Räder, H. J.; Müllen, K., Two-Dimensional Graphene Nanoribbons. *J. Am. Chem. Soc.* **2008,** *130*, 4216-4217.

(7) M. J. Frisch, G. W. T., H. B. Schlegel, G. E. Scuseria, ; M. A. Robb, J. R. C., G. Scalmani, V. Barone, ; G. A. Petersson, H. N., X. Li, M. Caricato, A. V. Marenich, ; J. Bloino, B. G. J., R. Gomperts, B. Mennucci, H. P. Hratchian, ; J. V. Ortiz, A. F. I., J. L. Sonnenberg, D. Williams-Young, ; F. Ding, F. L., F. Egidi, J. Goings, B. Peng, A. Petrone, ; T. Henderson, D. R., V. G. Zakrzewski, J. Gao, N. Rega, ; G. Zheng, W. L., M. Hada, M. Ehara, K. Toyota, R. Fukuda, ; J. Hasegawa, M. I., T. Nakajima, Y. Honda, O. Kitao, H. Nakai, ; T. Vreven, K. T., J. A. Montgomery, Jr., J. E. Peralta, ; F. Ogliaro, M. J. B., J. J. Heyd, E. N.

Brothers, K. N. Kudin, ; V. N. Staroverov, T. A. K., R. Kobayashi, J. Normand, ; K. Raghavachari, A. P. R., J. C. Burant, S. S. Iyengar, ; J. Tomasi, M. C., J. M. Millam, M. Klene, C. Adamo, R. Cammi, ; J. W. Ochterski, R. L. M., K. Morokuma, O. Farkas, ; J. B. Foresman, a. D. J. F., *Gaussian, Inc., Wallingford CT*, **2016**.
(8) Li, G.; Yoon, K.-Y.; Zhong, X.; Zhu, X.; Dong, G., Efficient Bottom-Up Preparation of Graphene Nanoribbons by Mild Suzuki–Miyaura Polymerization of Simple Triaryl Monomers. *Chem. Eur. J.* **2016,** *22*, 9116-9120.
(9) Momper, R.; Zhang, H.; Chen, S.; Halim, H.; Johannes, E.; Yordanov, S.; Braga, D.; Blülle, B.; Doblas, D.; Kraus, T.; Bonn, M.; Wang, H. I.; Riedinger, A., Kinetic Control over Self-Assembly of Semiconductor Nanoplatelets. *Nano Lett.* **2020,** *20*, 4102-4110.
(10) Zheng, W.; Bonn, M.; Wang, H. I., Photoconductivity Multiplication in Semiconducting Few-Layer $MoTe_2$. *Nano Lett.* **2020,** *20*, 5807-5813.
(11) Xu, F.; Yu, C.; Tries, A.; Zhang, H.; Kläui, M.; Basse, K.; Hansen, M. R.; Bilbao, N.; Bonn, M.; Wang, H. I.; Mai, Y., Tunable Superstructures of Dendronized Graphene Nanoribbons in Liquid Phase. *J. Am. Chem. Soc.* **2019,** *141*, 10972-10977.
(12) Niu, W.; Ma, J.; Soltani, P.; Zheng, W.; Liu, F.; Popov, A. A.; Weigand, J. J.; Komber, H.; Poliani, E.; Casiraghi, C.; Droste, J.; Hansen, M. R.; Osella, S.; Beljonne, D.; Bonn, M.; Wang, H. I.; Feng, X.; Liu, J.; Mai, Y., A Curved Graphene Nanoribbon with Multi-Edge Structure and High Intrinsic Charge Carrier Mobility. *J. Am. Chem. Soc.* **2020,** *142*, 18293-18298.
(13) Ivanov, I.; Hu, Y.; Osella, S.; Beser, U.; Wang, H. I.; Beljonne, D.; Narita, A.; Müllen, K.; Turchinovich, D.; Bonn, M., Role of Edge Engineering in Photoconductivity of Graphene Nanoribbons. *J. Am. Chem. Soc.* **2017,** *139*, 7982-7988.
(14) Chen, Z.; Wang, H. I.; Teyssandier, J.; Mali, K. S.; Dumslaff, T.; Ivanov, I.; Zhang, W.; Ruffieux, P.; Fasel, R.; Räder, H. J.; Turchinovich, D.; De Feyter, S.; Feng, X.; Kläui, M.; Narita, A.; Bonn, M.; Müllen, K., Chemical Vapor Deposition Synthesis and Terahertz Photoconductivity of Low-Band-Gap N = 9 Armchair Graphene Nanoribbons. *J. Am. Chem. Soc.* **2017,** *139*, 3635-3638.
